# Supplementary figures and images for: Actin-regulated Siglec-1 nanoclustering influences HIV-1 capture and virus-containing compartment formation in dendritic cells
Source: eLife. 2023 Mar 20;12:e78836. doi: 10.7554/eLife.78836 (PMC10065798; doi:10.7554/eLife.78836)

Figure 1

A

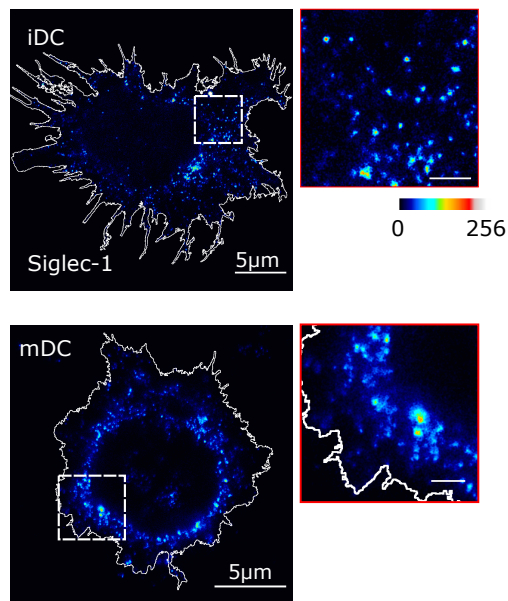

B

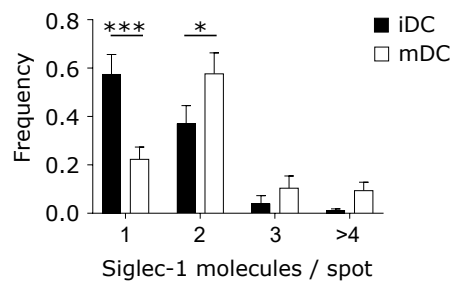

C

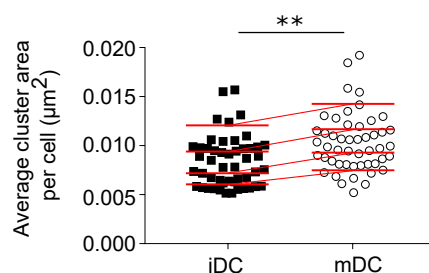

D

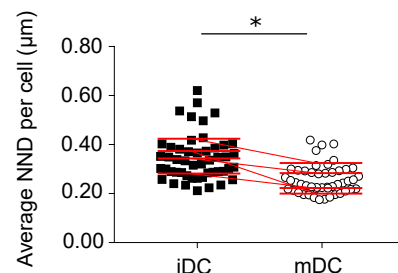

E

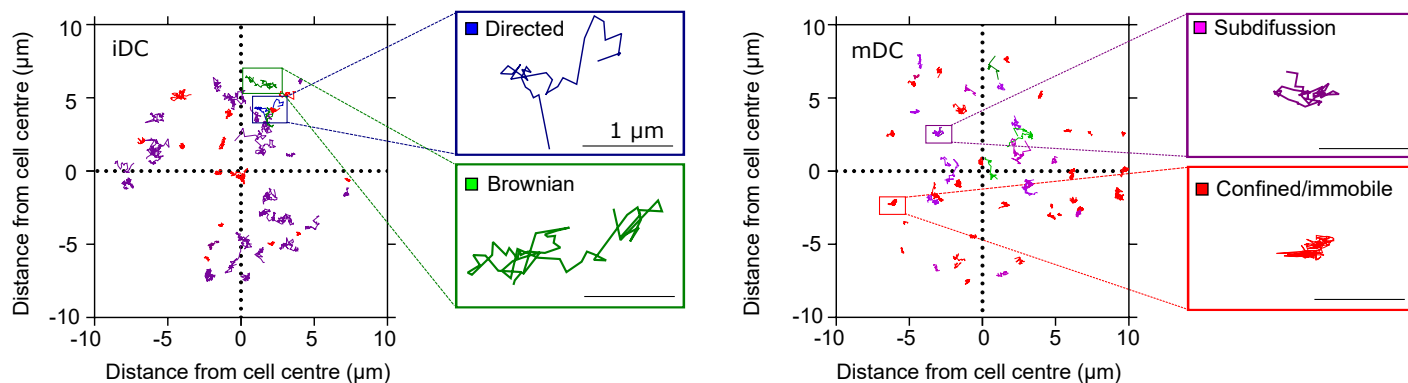

F

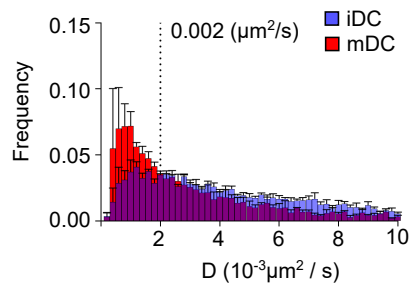

G

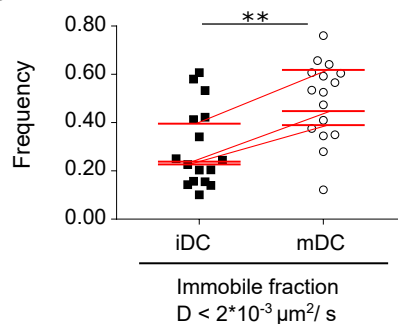

H

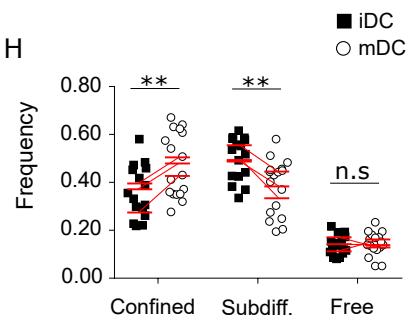

I

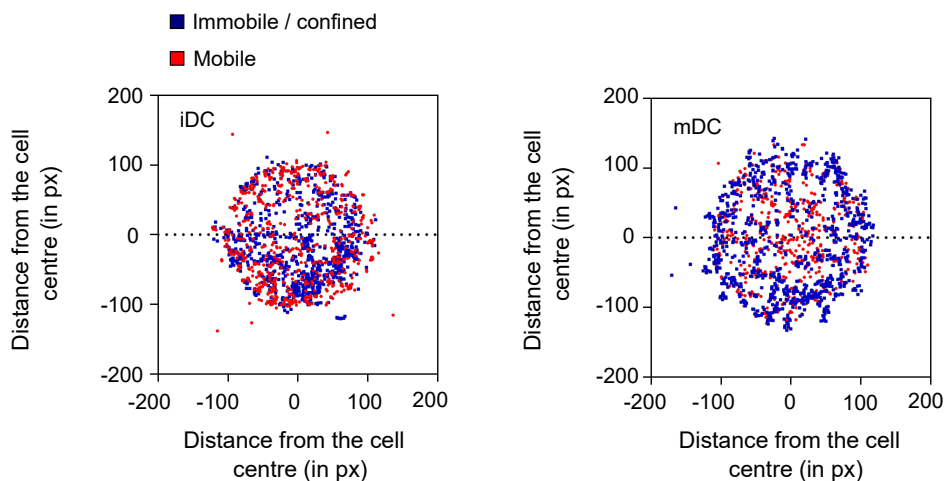

J

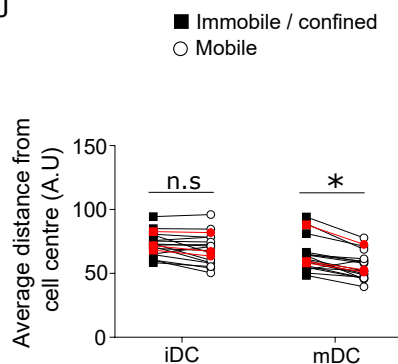

Supplement: Figure 1—source data 1. [file elife-78836-fig1-data1.zip › Figure 1-source data 1/Figure 1.pdf]

Uncropped gel image of Figure 1-figure supplement 1E

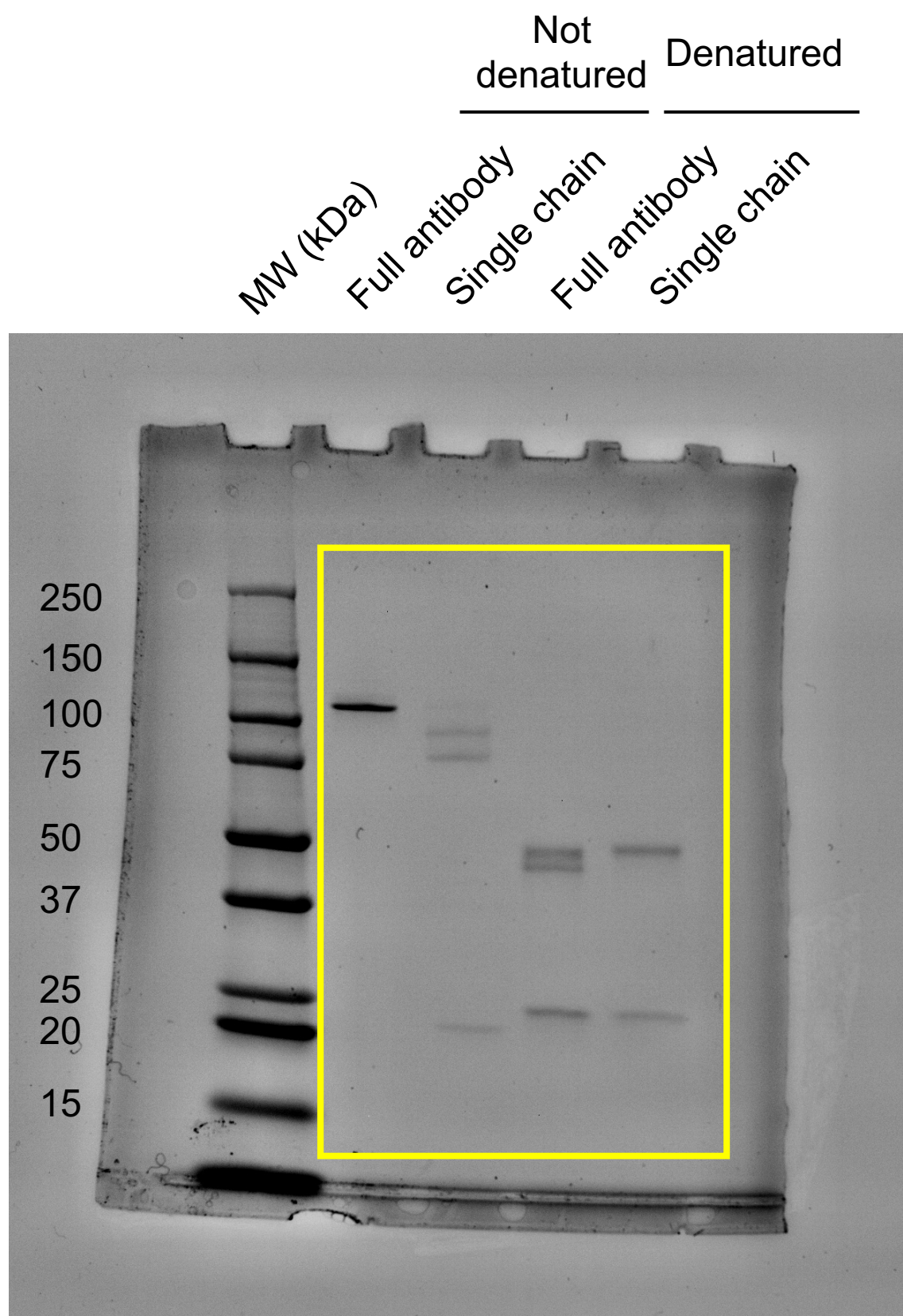

Supplement: Figure 1—figure supplement 1—source data 1. [file elife-78836-fig1-figsupp1-data1.zip › Fig1-figsup1e-source.pdf]

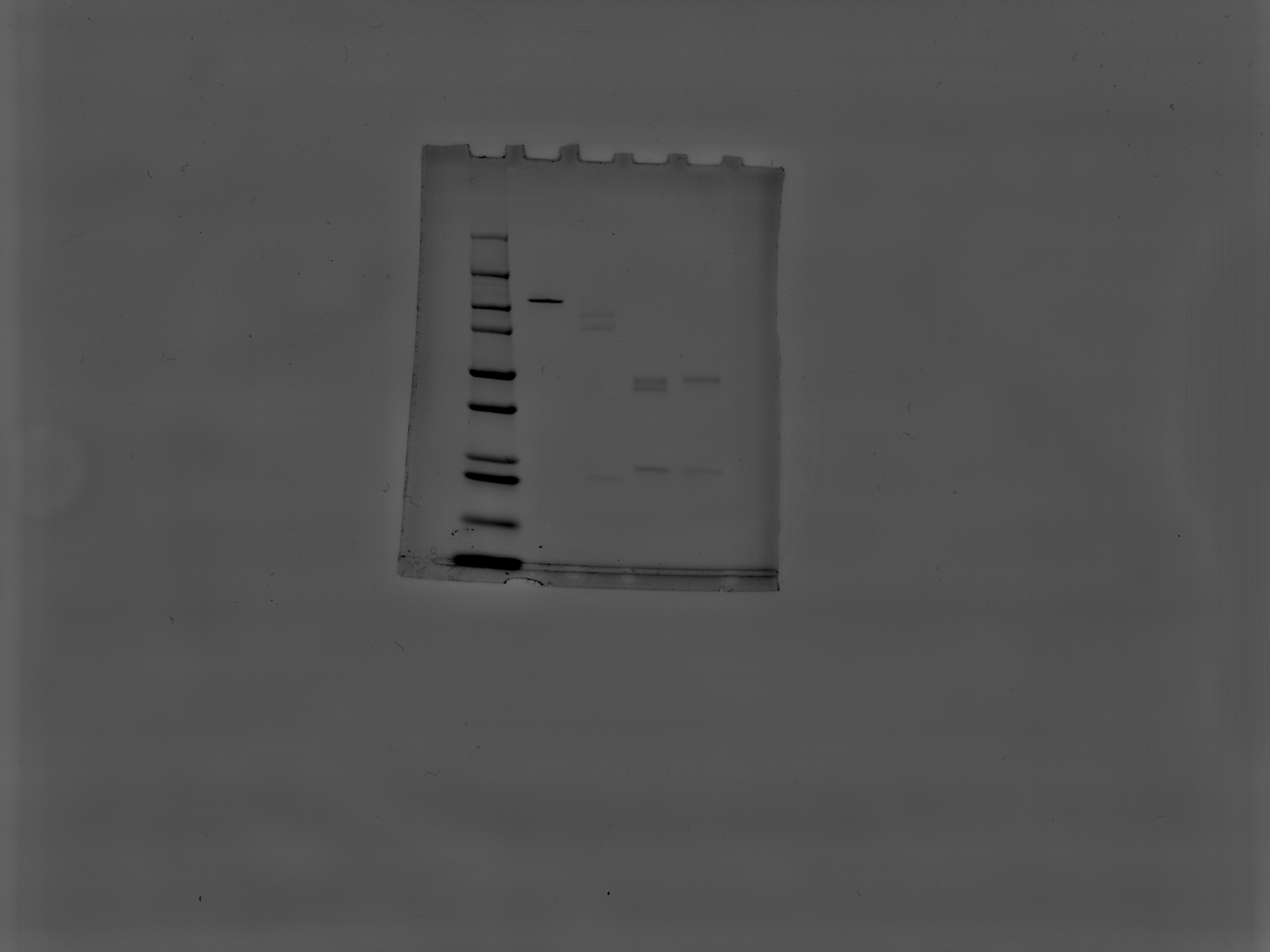

Supplement: Figure 1—figure supplement 1—source data 1. [file elife-78836-fig1-figsupp1-data1.zip › Siglec1_singlechains_2_pub.tif]

**Figure 1-figure supplement 1**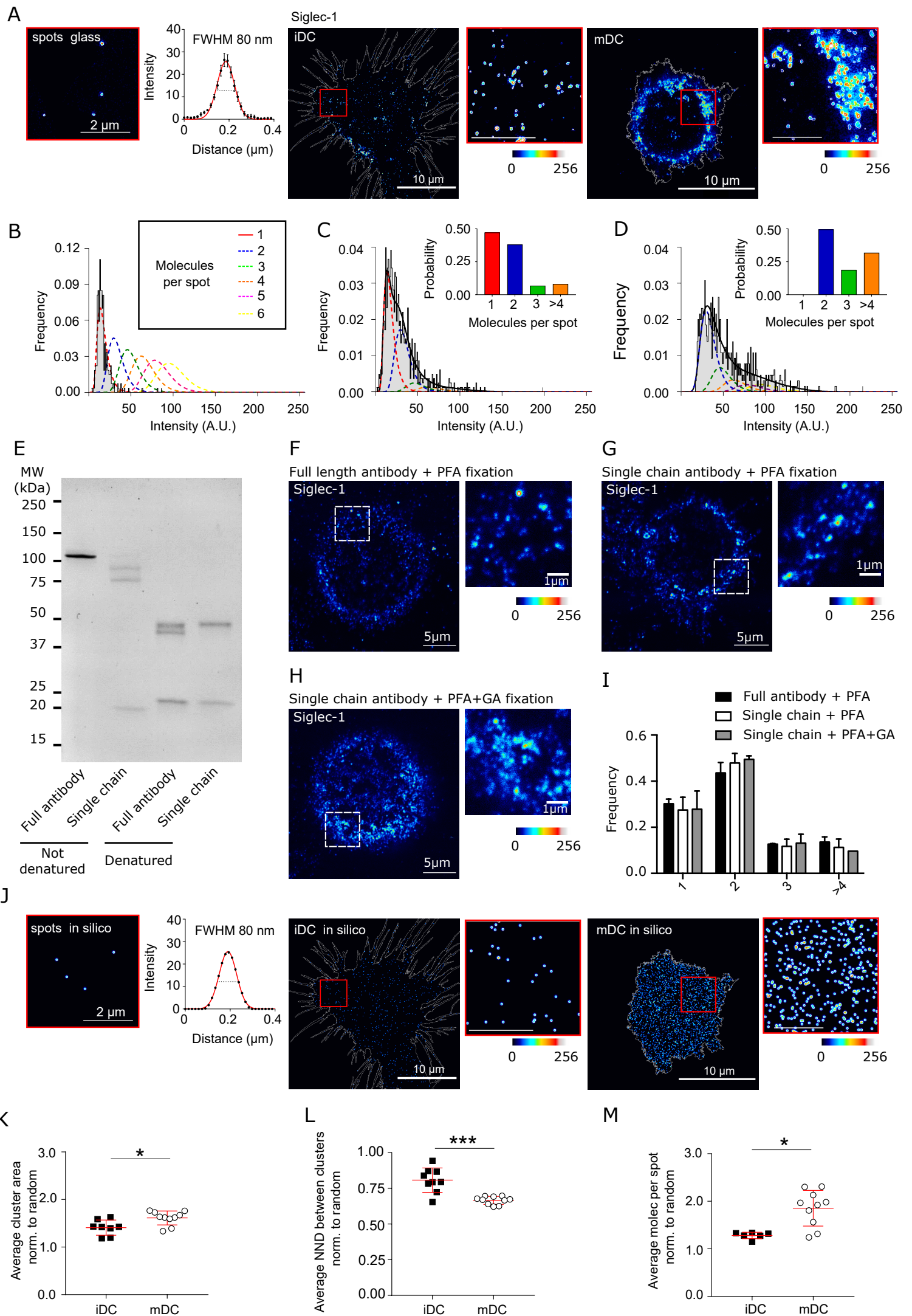

Supplement: Figure 1—figure supplement 1—source data 2. [file elife-78836-fig1-figsupp1-data2.zip › Figure 1-figure supplement 1-source data 2/Figure 1-figure supplement 1.pdf]

Figure 2

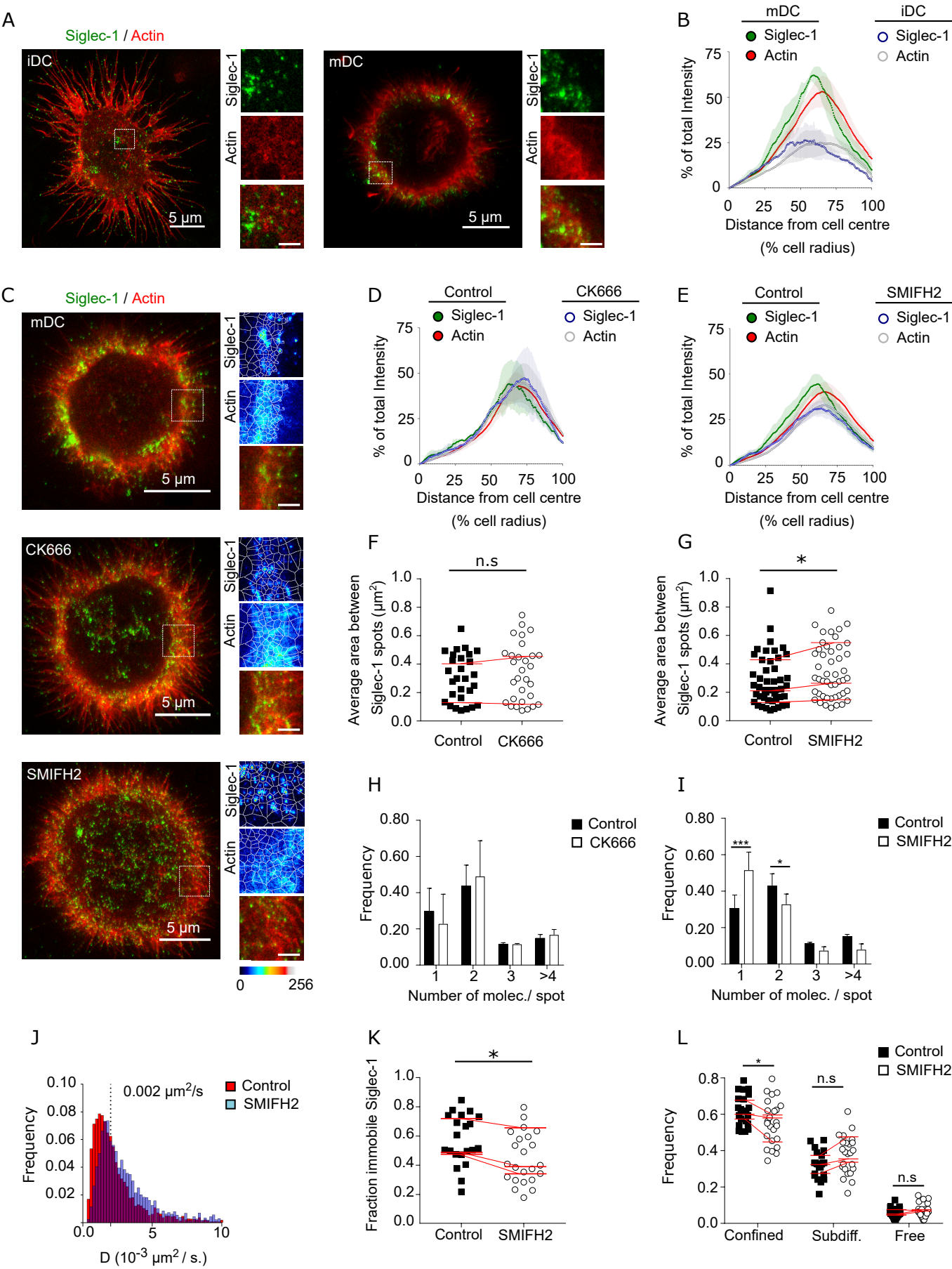

Supplement: Figure 2—source data 1. [file elife-78836-fig2-data1.zip › Figure 2-source data 1/Figure 2.pdf]

Figure 2-figure supplement 1

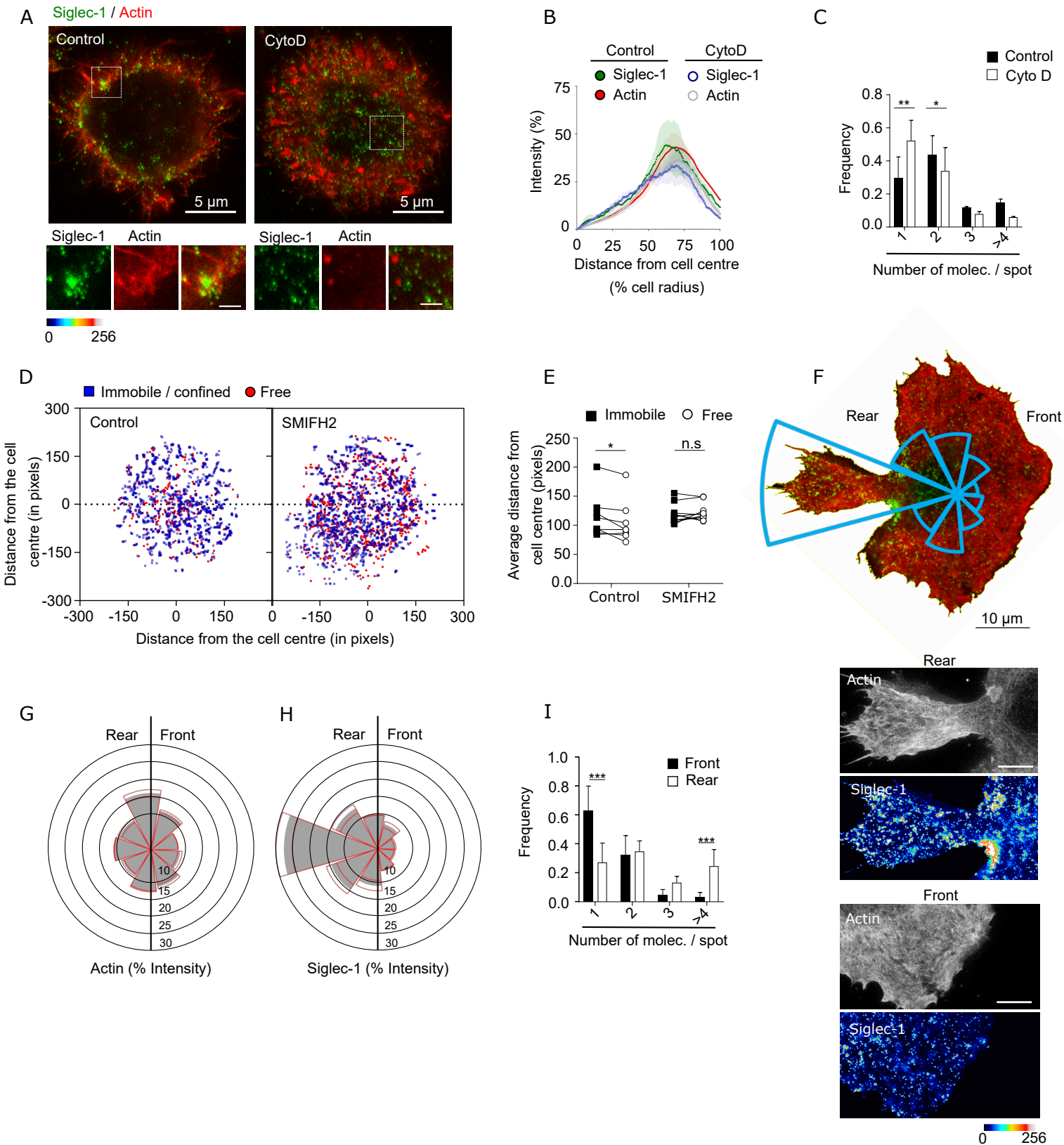

Supplement: Figure 2—figure supplement 1—source data 1. [file elife-78836-fig2-figsupp1-data1.zip › Figure 2-figure supplement 1-source data 1/Figure 2-figure supplement 1.pdf]

Figure 3

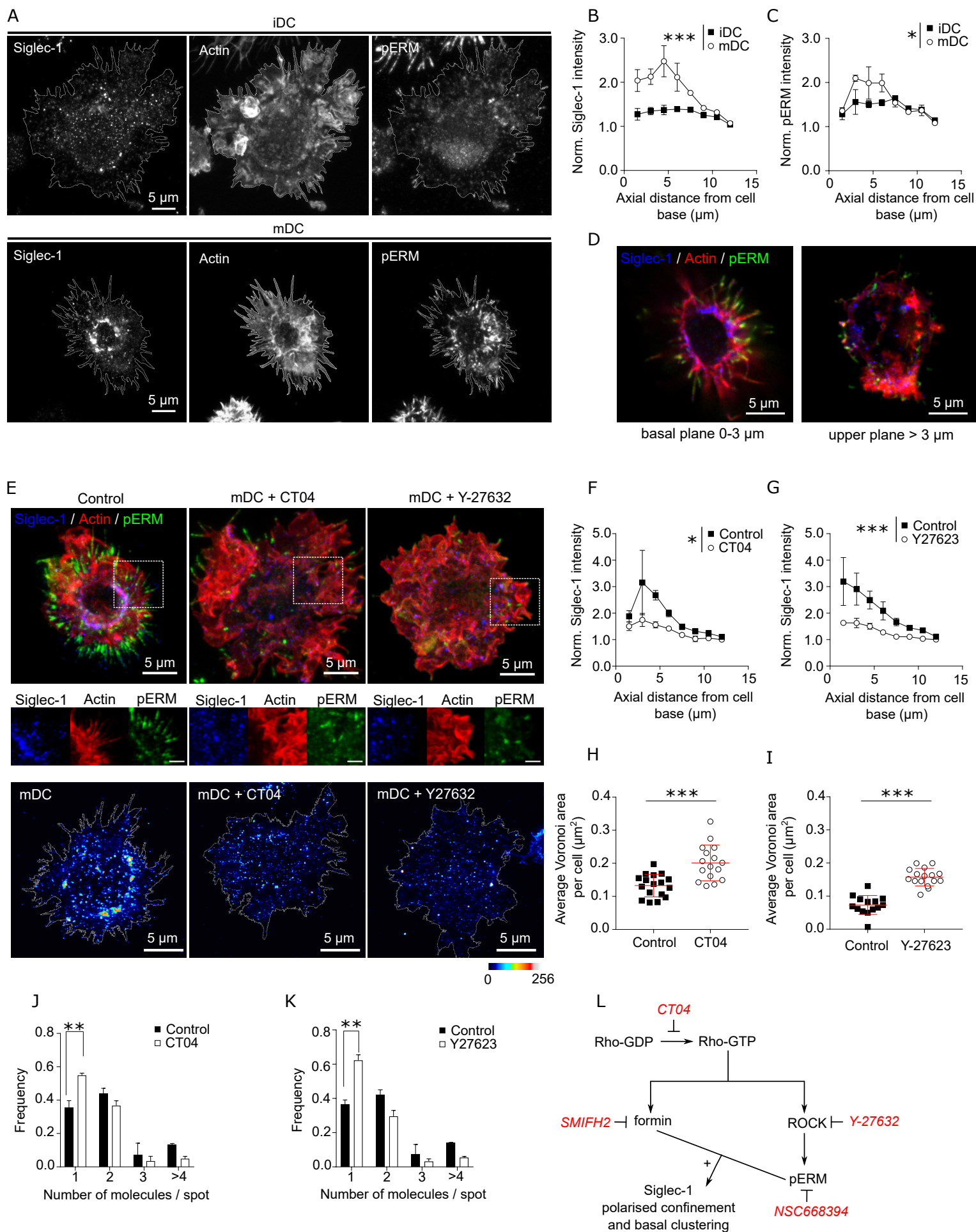

Supplement: Figure 3—source data 1. [file elife-78836-fig3-data1.zip › Figure 3-source data 1/Figure 3.pdf]

**Figure 3-figure supplement 1**

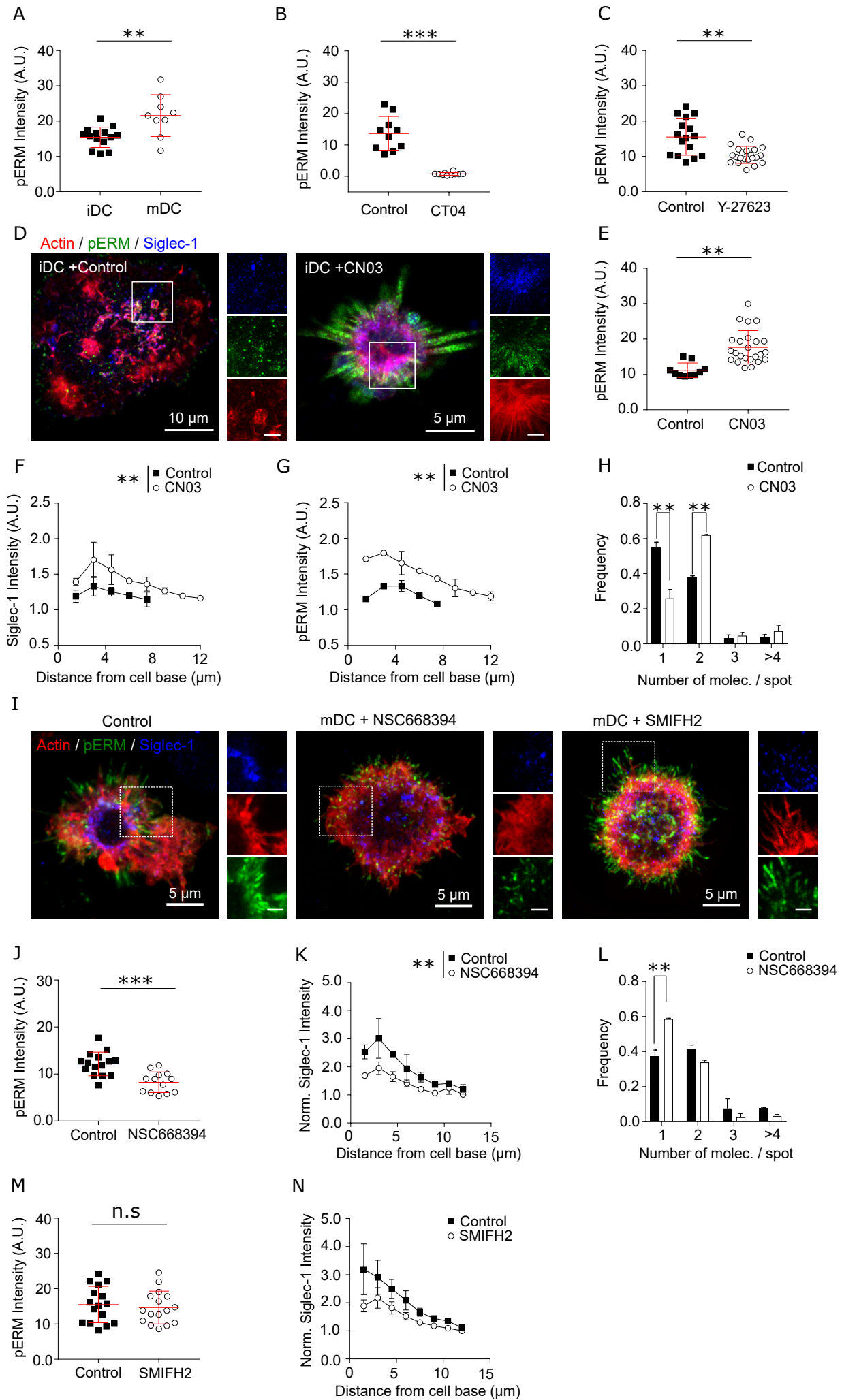

Supplement: Figure 3—figure supplement 1—source data 1. [file elife-78836-fig3-figsupp1-data1.zip › Figure 3-figure supplement 1-source data 1/Figure 3-figure supplement 1.pdf]

Figure 4

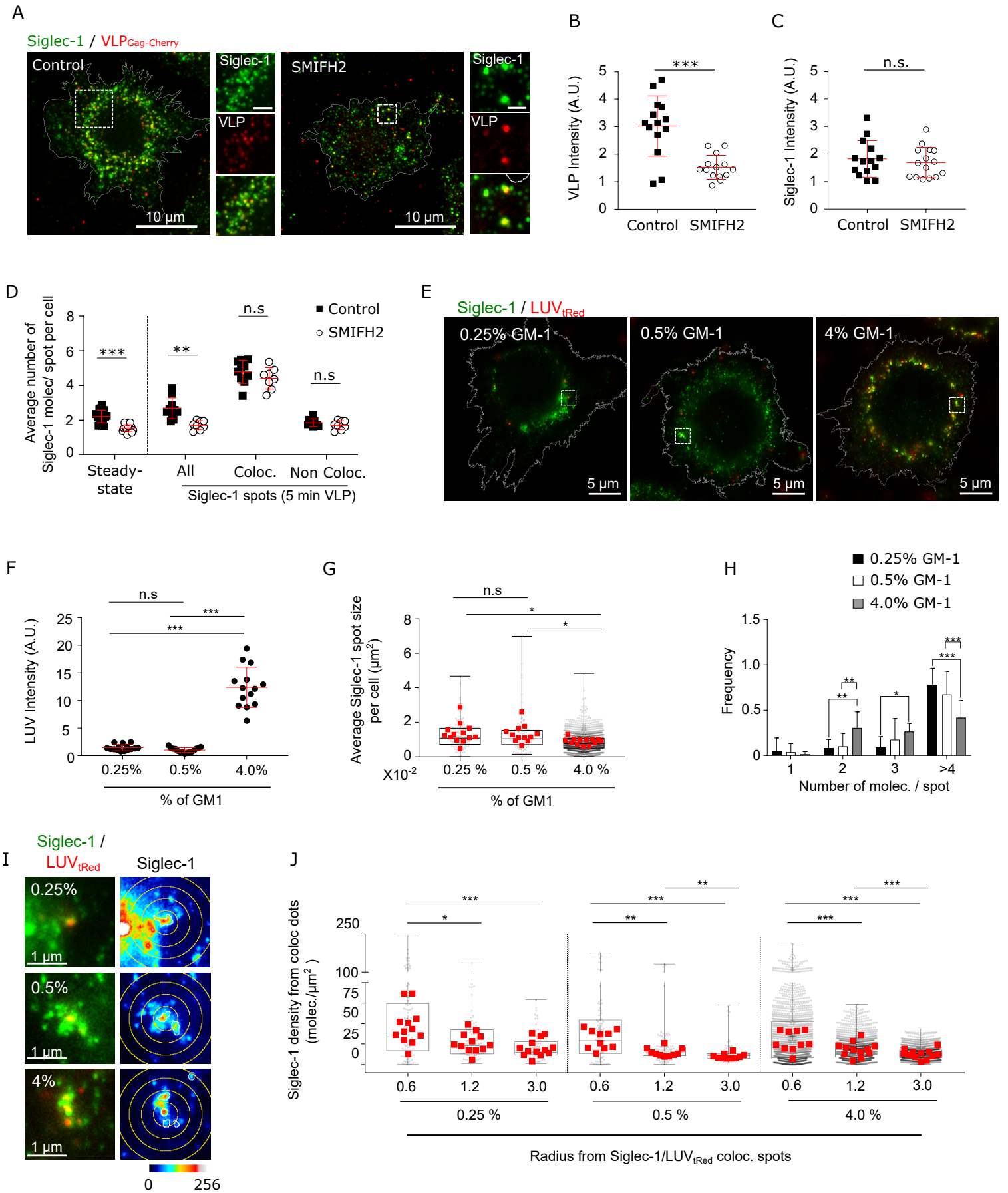

Supplement: Figure 4—source data 1. [file elife-78836-fig4-data1.zip › Figure 4-source data 1/Figure 4.pdf]

Figure 4-figure supplement 1

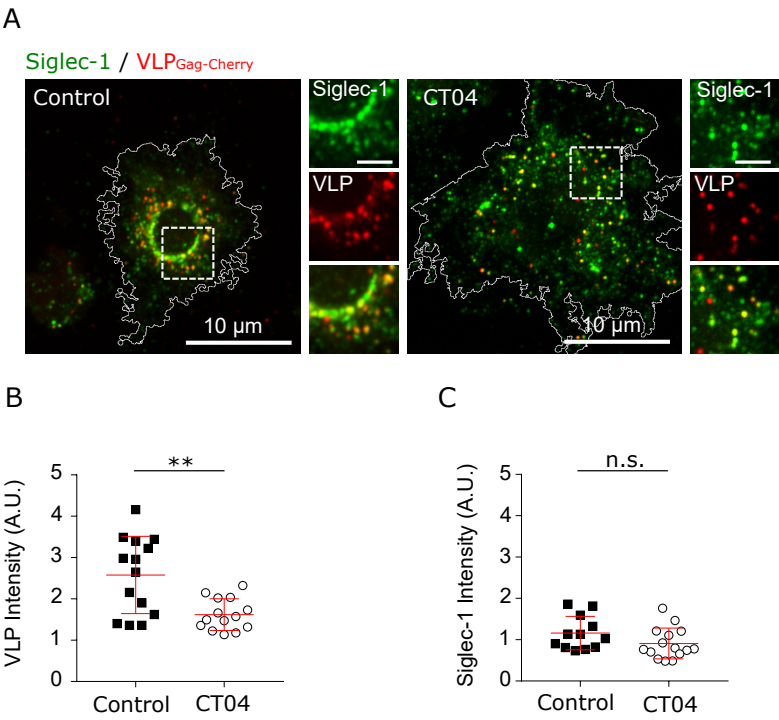

Supplement: Figure 4—figure supplement 1—source data 1. [file elife-78836-fig4-figsupp1-data1.zip › Figure 4-figure supplement 1-source data 1/Figure 4-figure supplement 1.pdf]

Figure 5

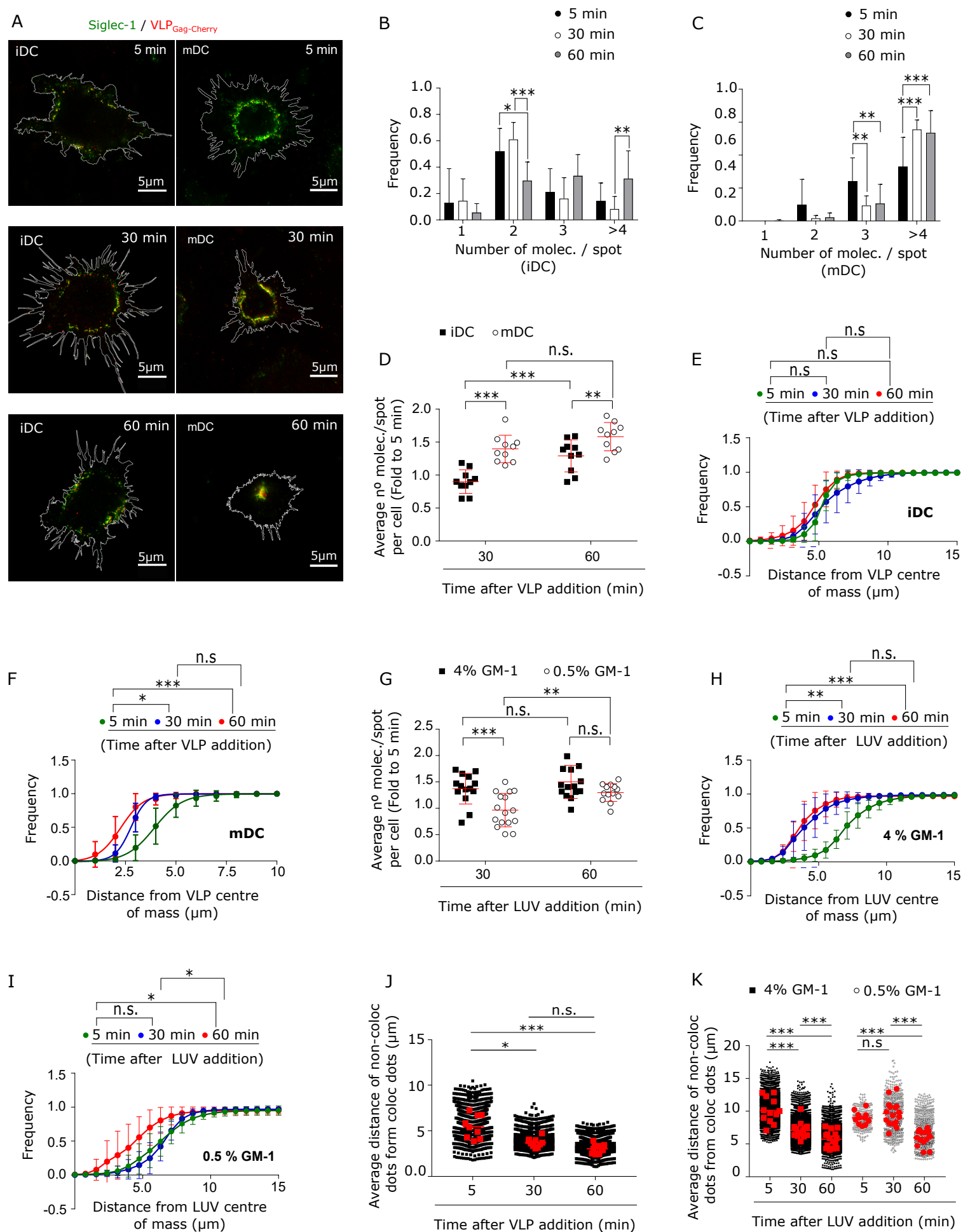

Supplement: Figure 5—source data 1. [file elife-78836-fig5-data1.zip › Figure 5-source data/Figure 5.pdf]

**Figure 5-figure supplement 1**

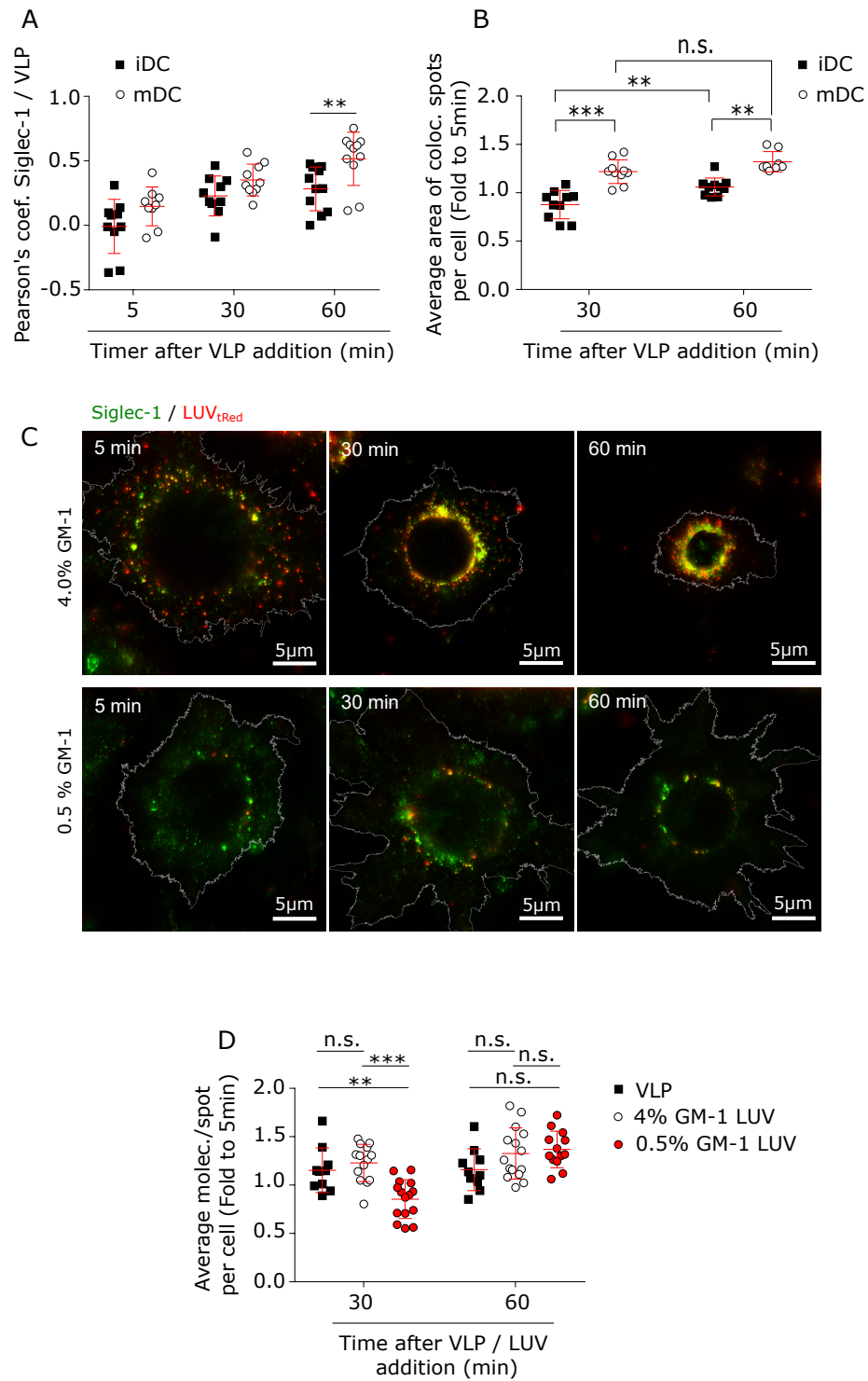

Supplement: Figure 5—figure supplement 1—source data 1. [file elife-78836-fig5-figsupp1-data1.zip › Figure 5-figure supplement 1-source data 1/Figure 5-figure supplement 1.pdf]

Figure 6

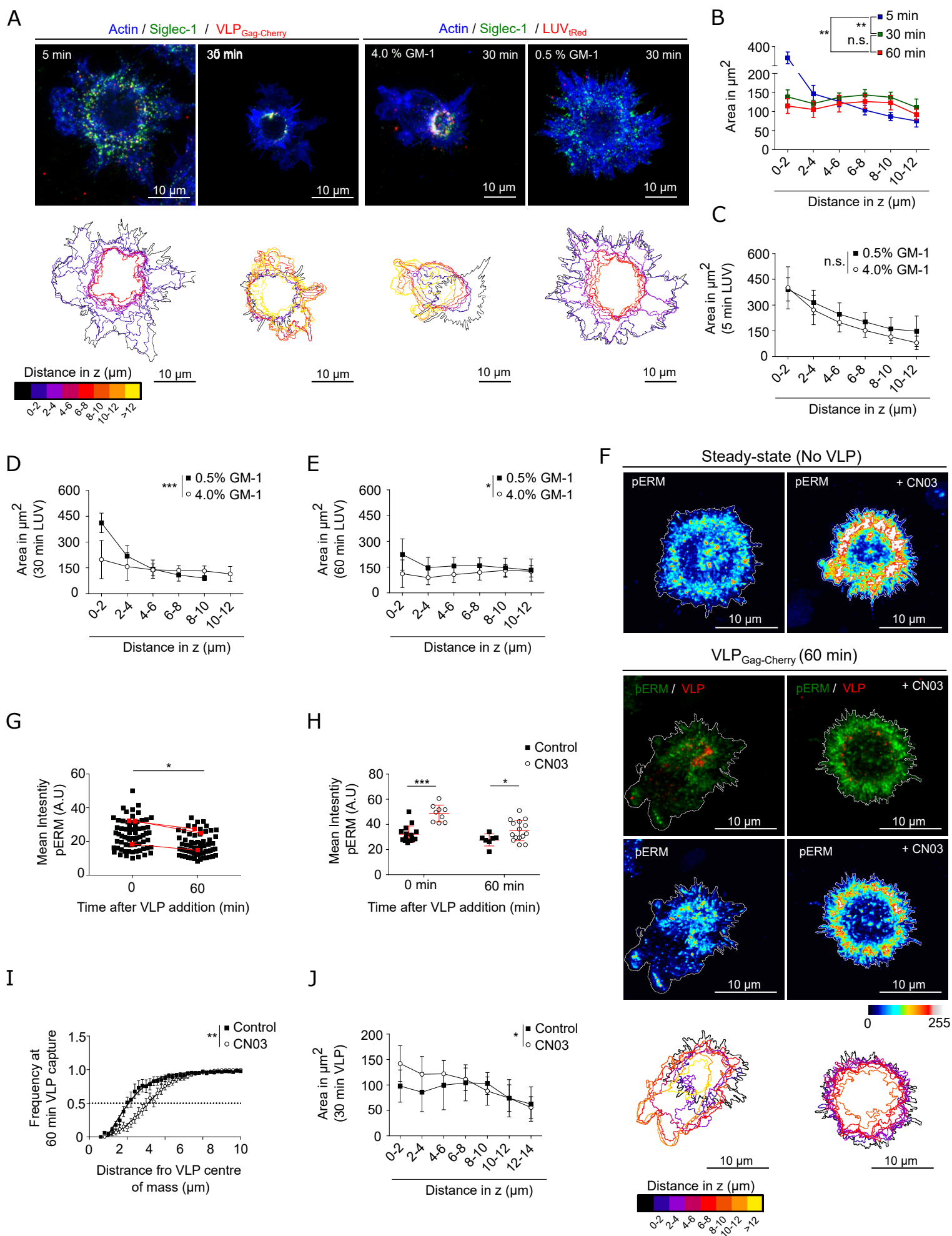

Supplement: Figure 6—source data 1. [file elife-78836-fig6-data1.zip › Figure 6-source data/Figure 6.pdf]

Figure 6-figure supplement 1

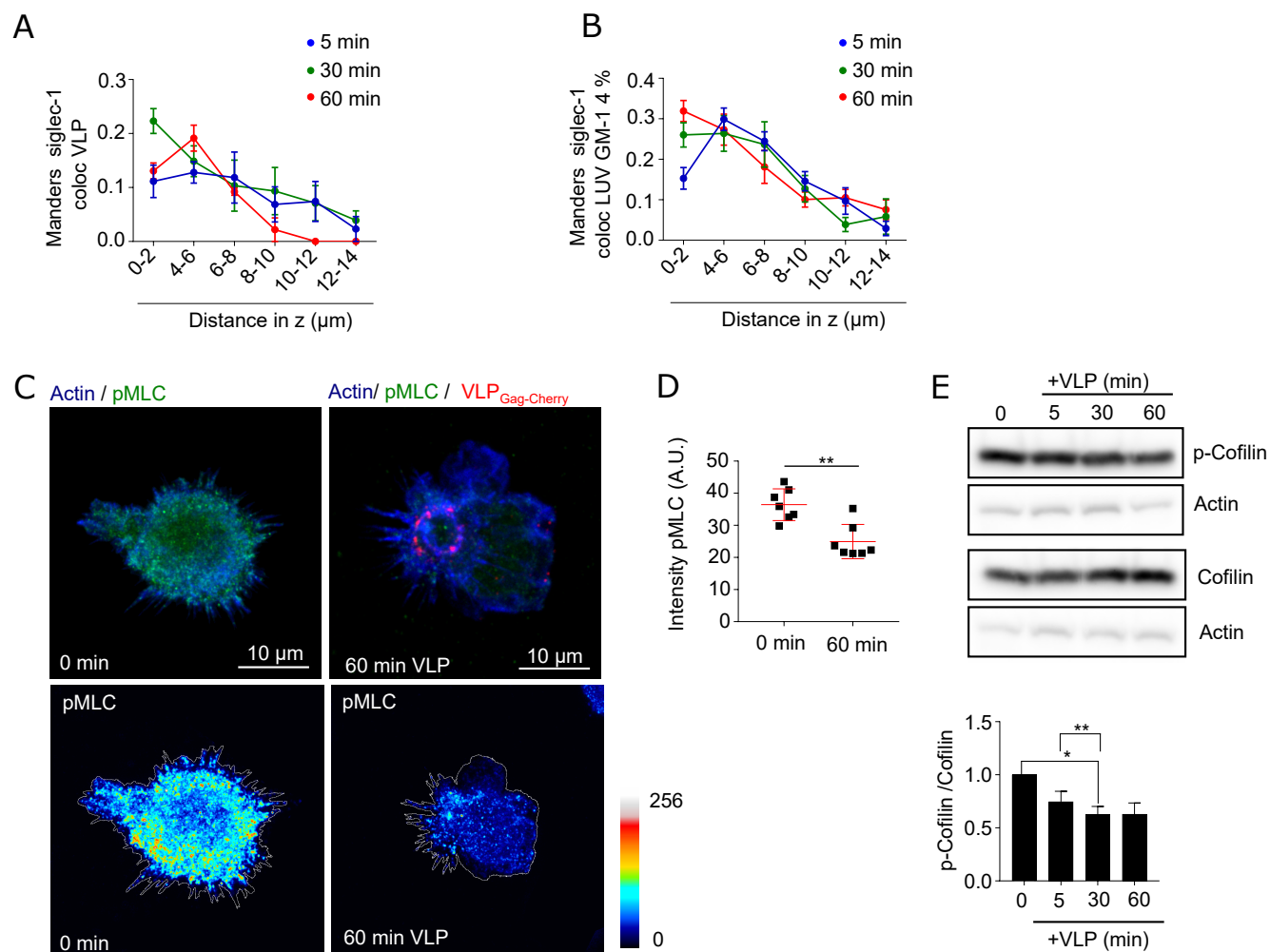

Supplement: Figure 6—figure supplement 1—source data 1. [file elife-78836-fig6-figsupp1-data1.zip › Figure 6-figure supplement 1-source data 1/Figure 6-figure supplement 1.pdf]

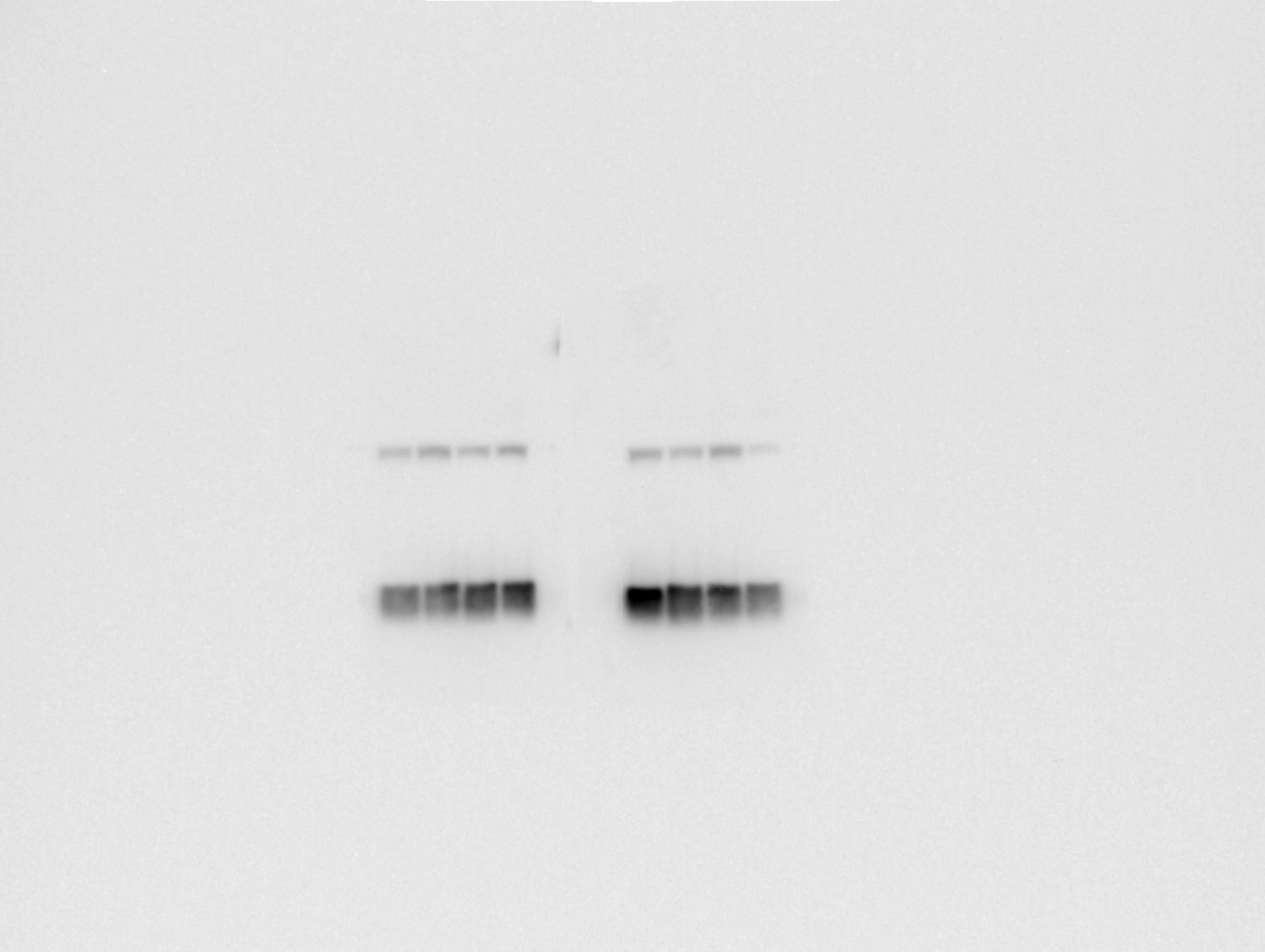

Supplement: Figure 6—figure supplement 1—source data 2. [file elife-78836-fig6-figsupp1-data2.zip › experiment 1/mDC cofilin and pcofilin and actin_pub.tif]

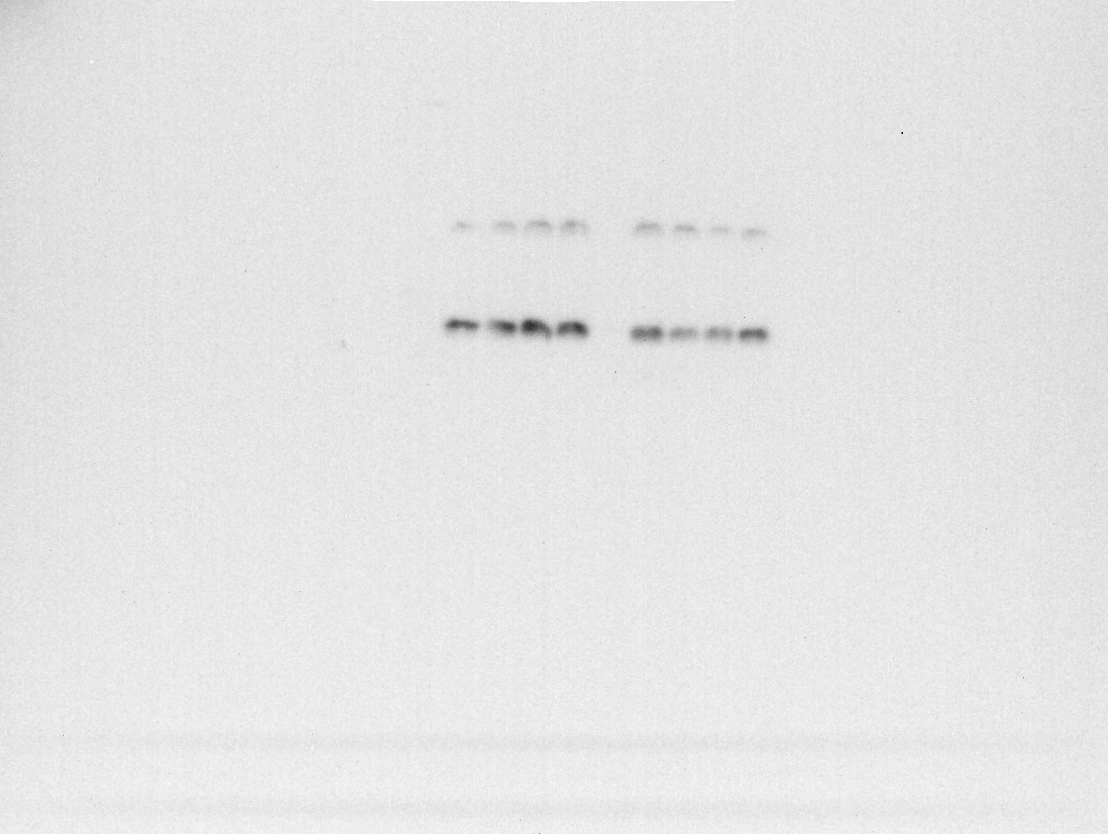

Supplement: Figure 6—figure supplement 1—source data 2. [file elife-78836-fig6-figsupp1-data2.zip › Experiment 2/Cofilin and p-Cofilin.tif]

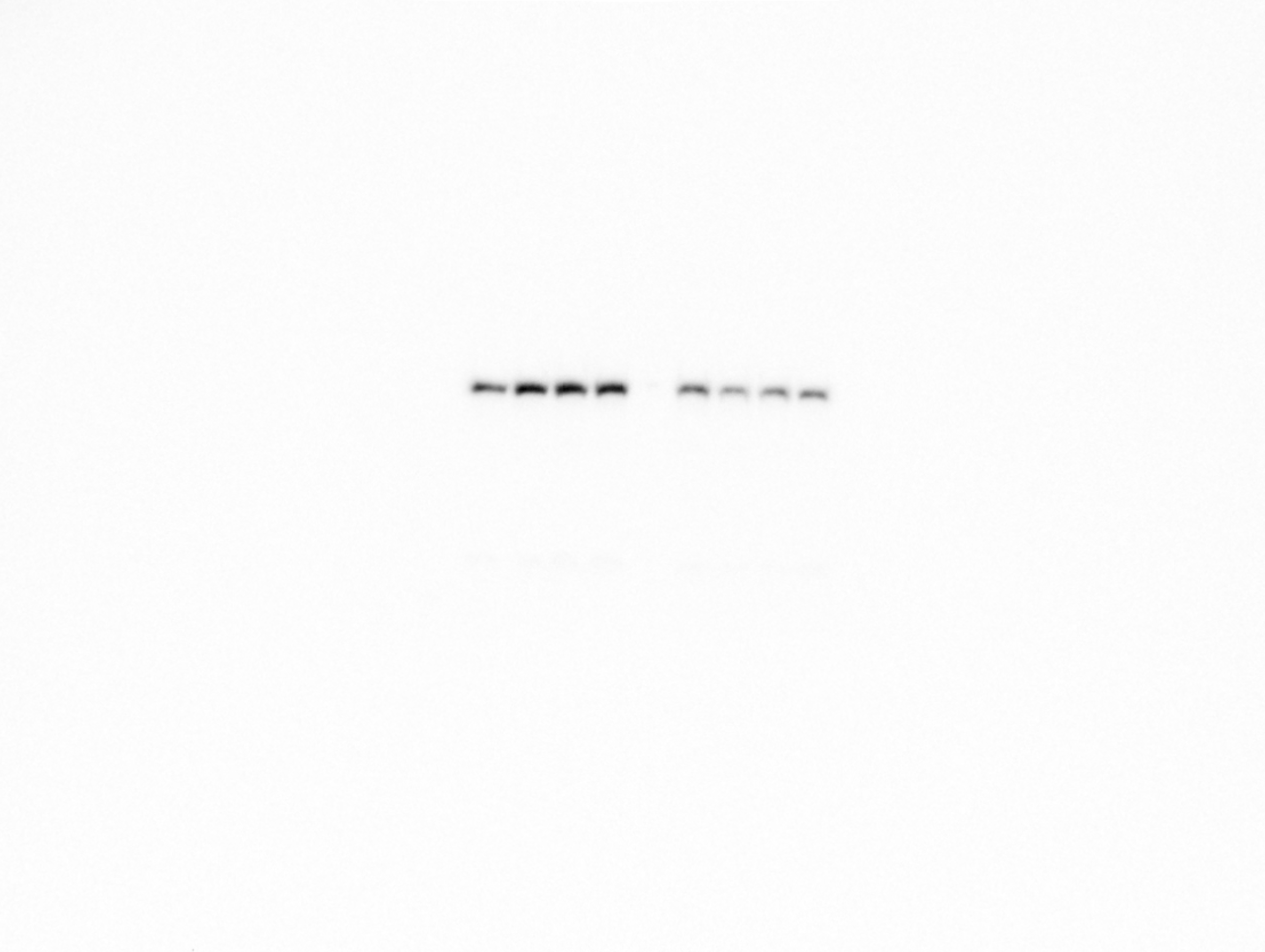

Supplement: Figure 6—figure supplement 1—source data 2. [file elife-78836-fig6-figsupp1-data2.zip › Experiment 2/actin.tif]

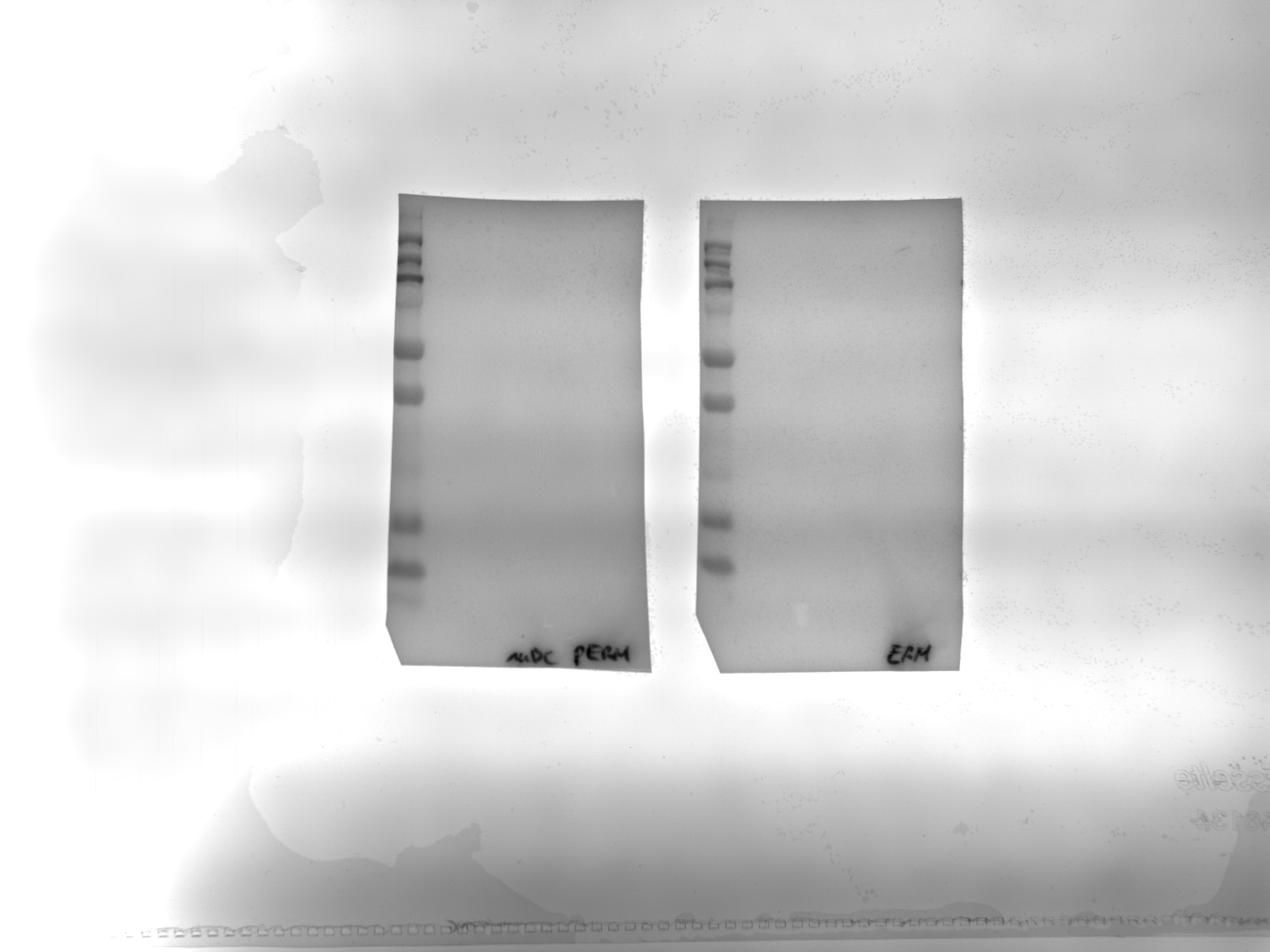

Supplement: Figure 6—figure supplement 1—source data 2. [file elife-78836-fig6-figsupp1-data2.zip › Experiment 3/mDC cofilin and pcofilin membrane.tif]

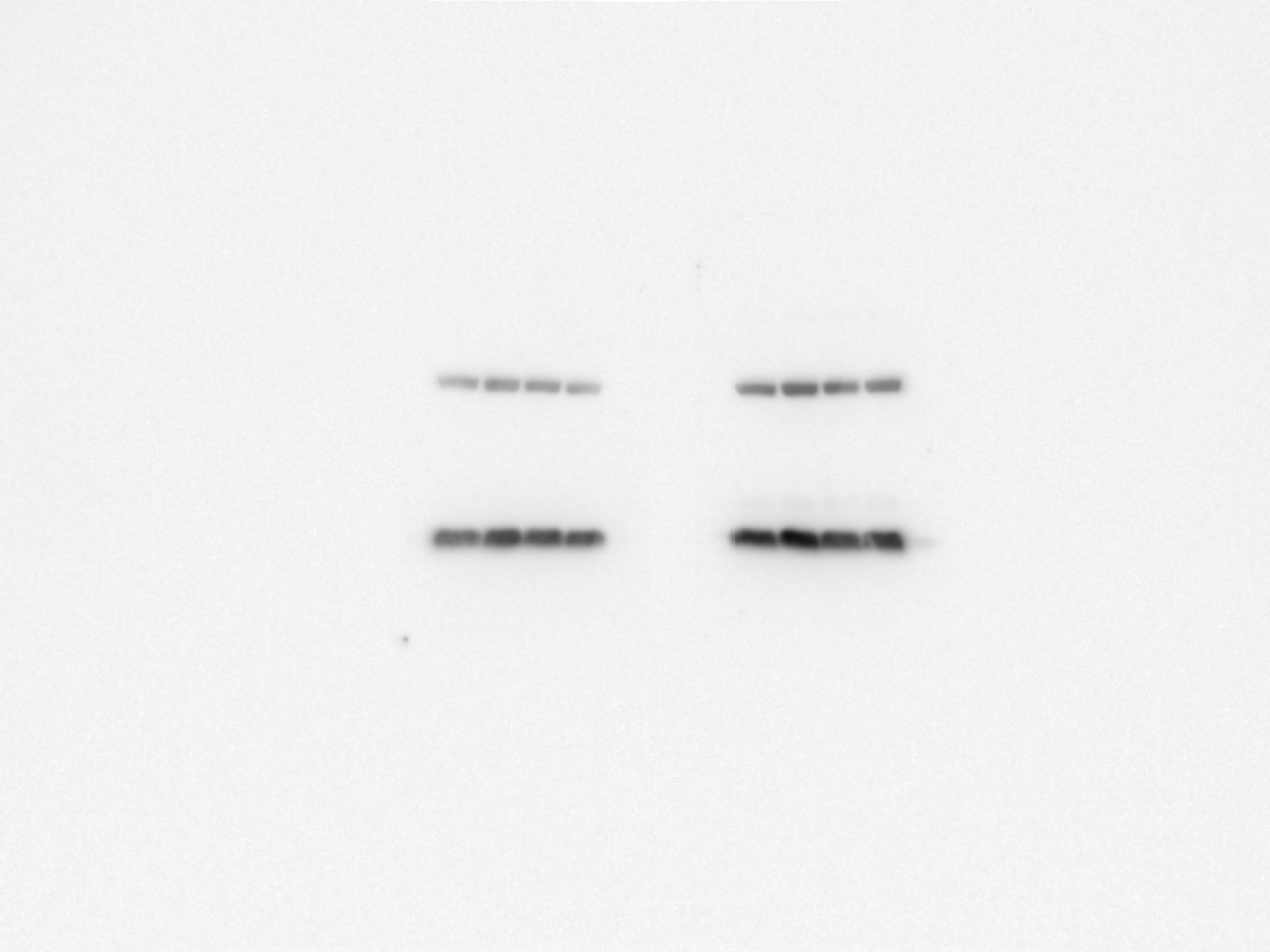

Supplement: Figure 6—figure supplement 1—source data 2. [file elife-78836-fig6-figsupp1-data2.zip › Experiment 3/cofilin and pcofilin.tif]

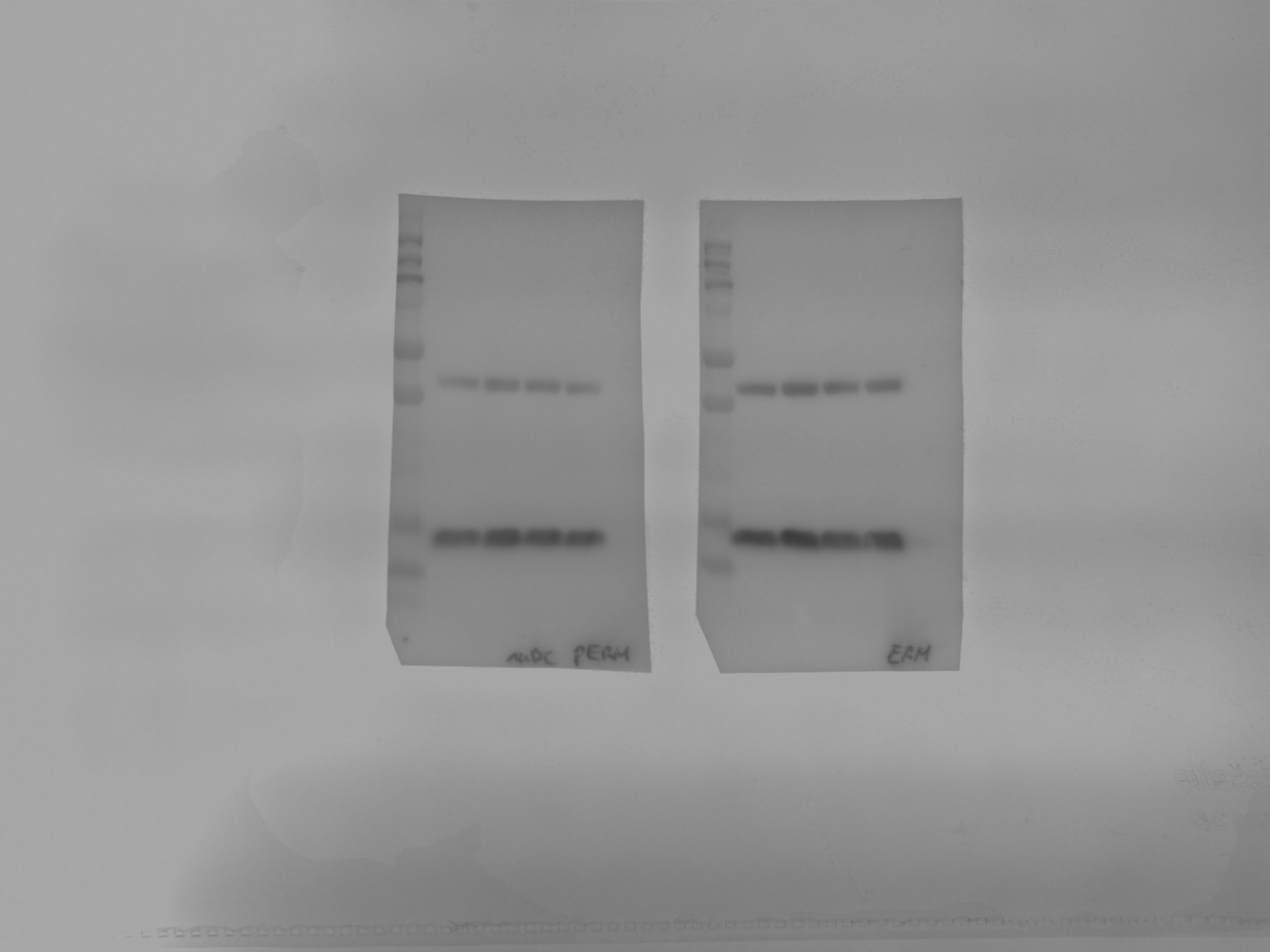

Supplement: Figure 6—figure supplement 1—source data 2. [file elife-78836-fig6-figsupp1-data2.zip › Experiment 3/Composite (RGB).tif]

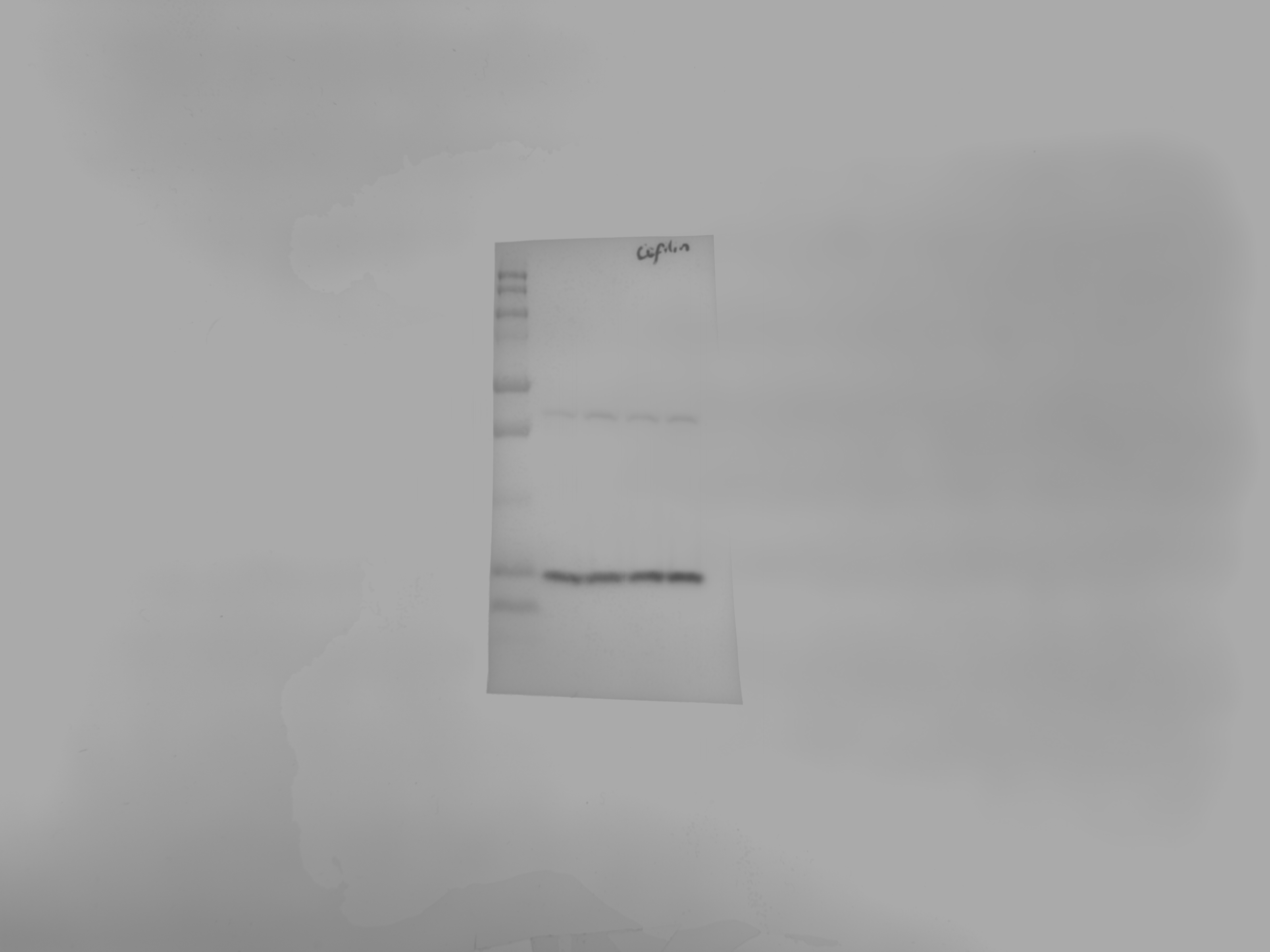

Supplement: Figure 6—figure supplement 1—source data 2. [file elife-78836-fig6-figsupp1-data2.zip › example image experiment 4/Composite cofilin.tif]

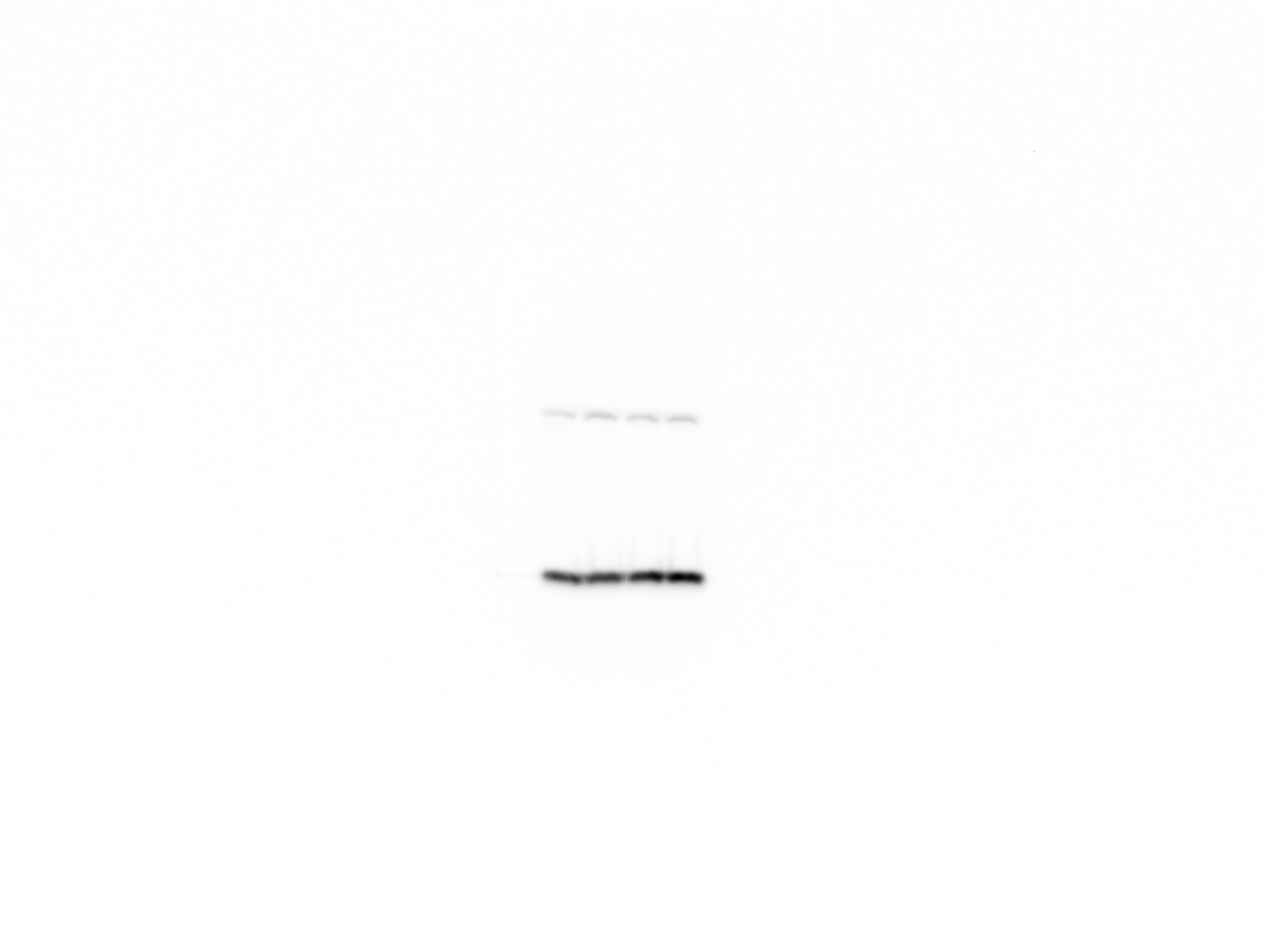

Supplement: Figure 6—figure supplement 1—source data 2. [file elife-78836-fig6-figsupp1-data2.zip › example image experiment 4/cofilin1_pub-2.tif]

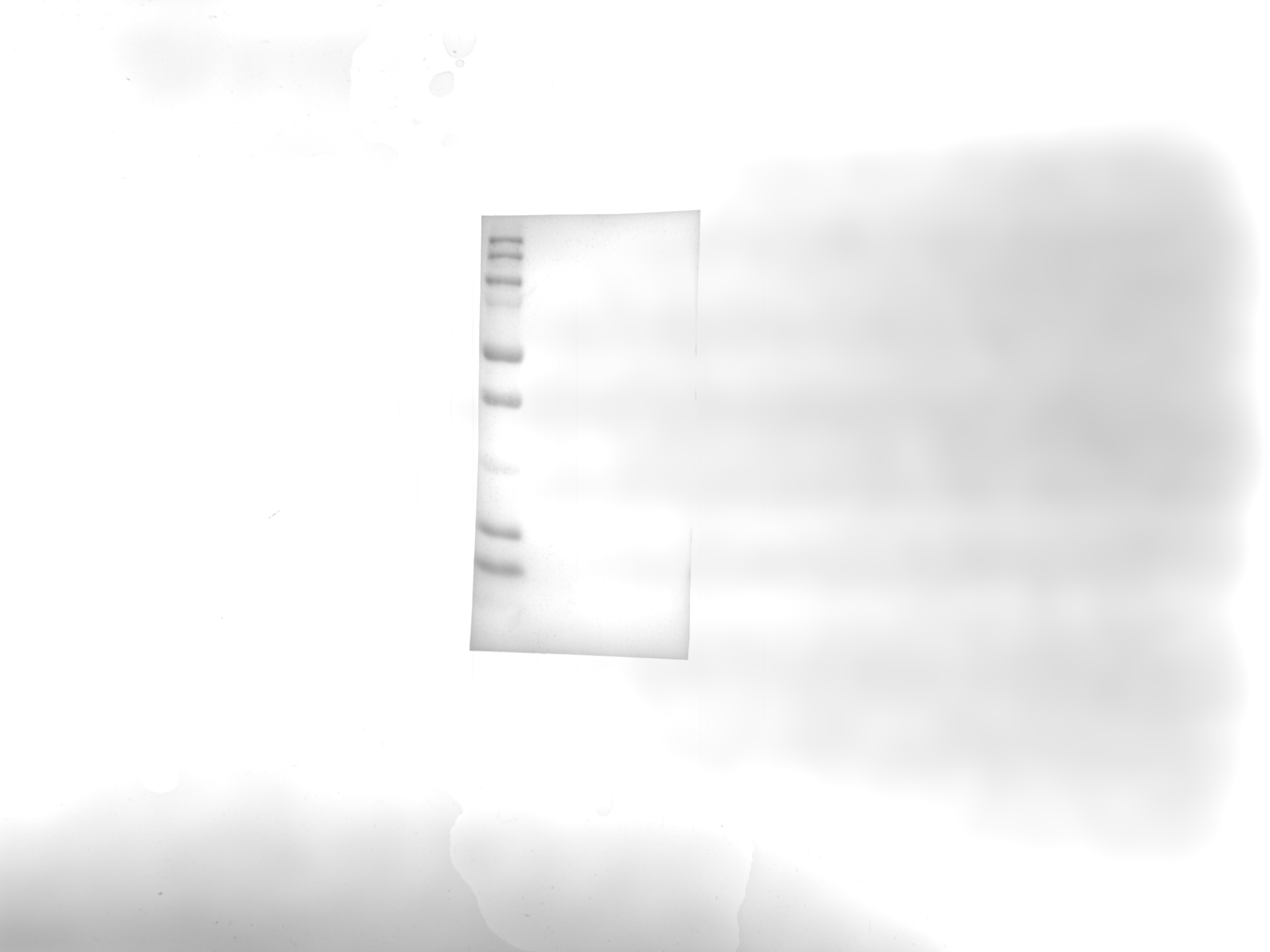

Supplement: Figure 6—figure supplement 1—source data 2. [file elife-78836-fig6-figsupp1-data2.zip › example image experiment 4/pcofilin1 membrane.tif]

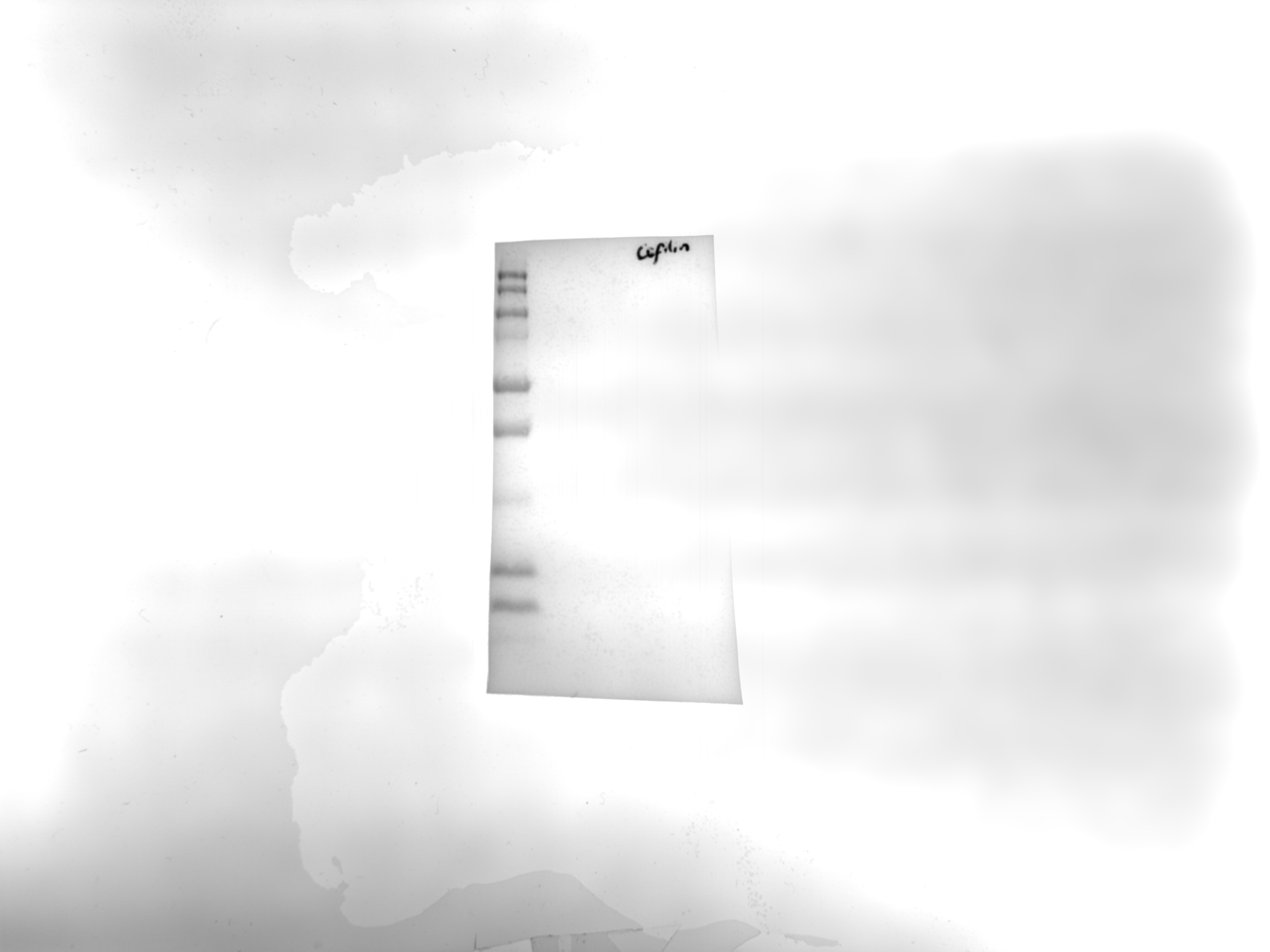

Supplement: Figure 6—figure supplement 1—source data 2. [file elife-78836-fig6-figsupp1-data2.zip › example image experiment 4/cofilin membrane_pub.tif]

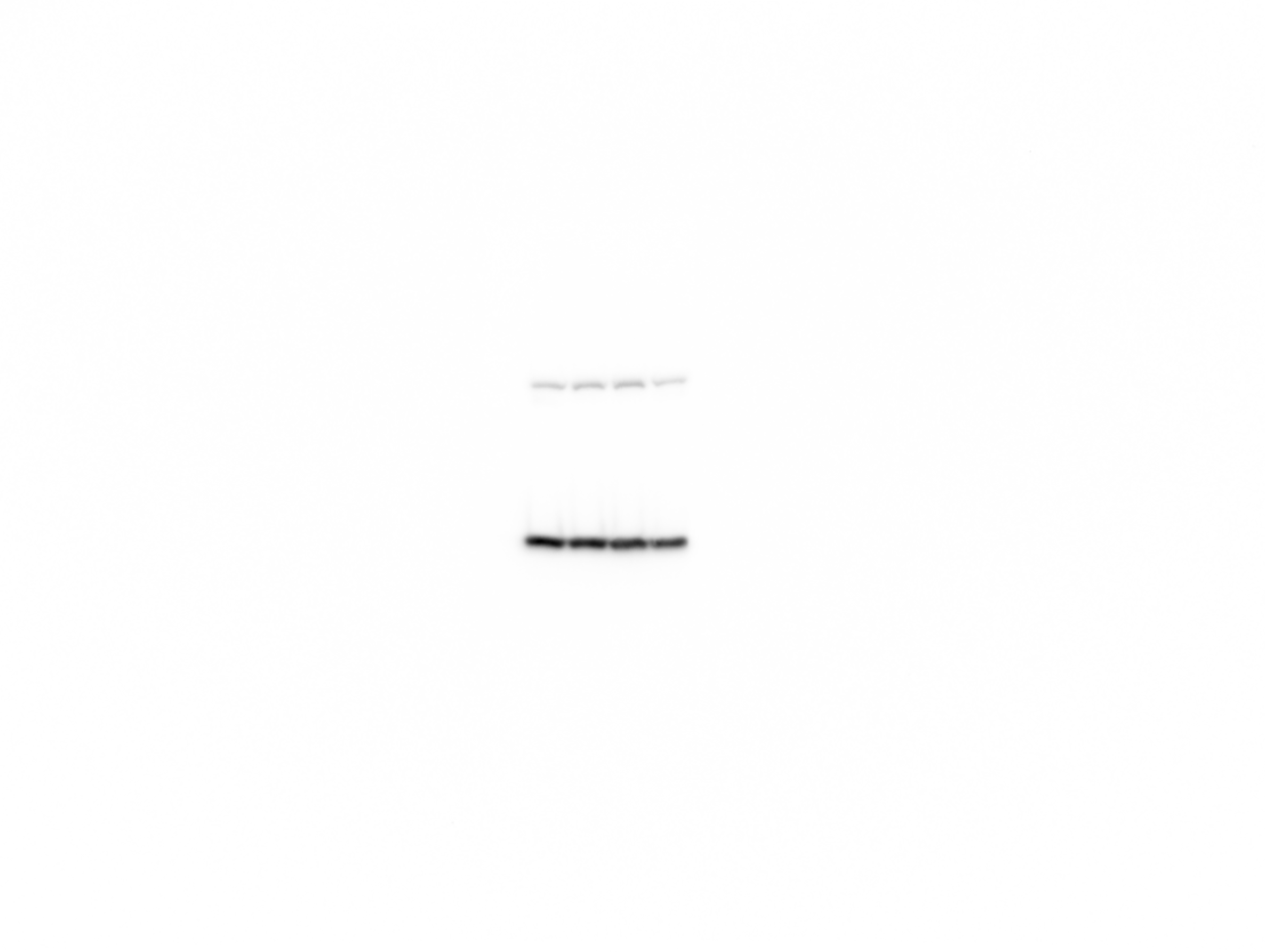

Supplement: Figure 6—figure supplement 1—source data 2. [file elife-78836-fig6-figsupp1-data2.zip › example image experiment 4/pcofilin1_pub.tif]

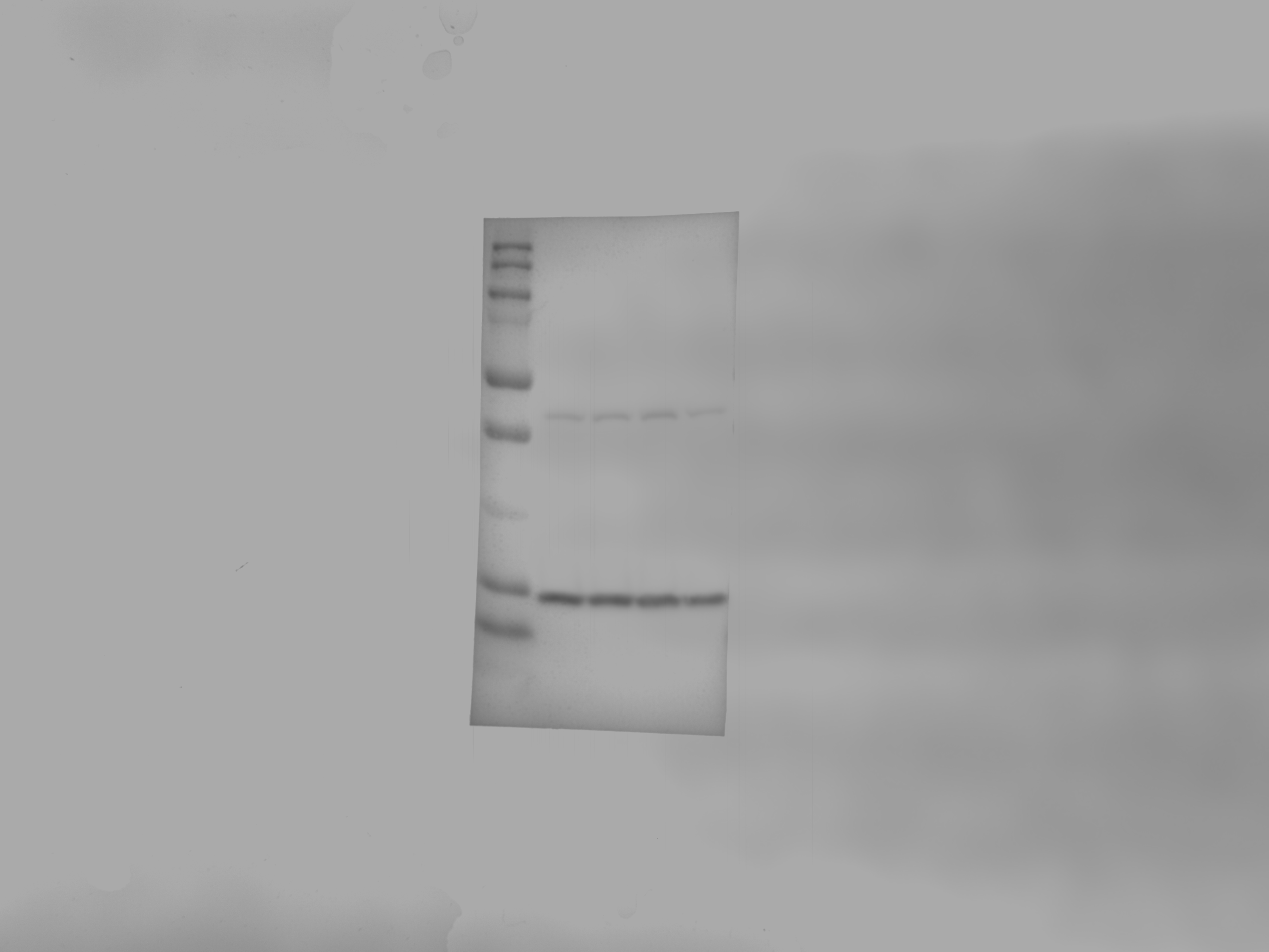

Supplement: Figure 6—figure supplement 1—source data 2. [file elife-78836-fig6-figsupp1-data2.zip › example image experiment 4/Composite pcofilin.tif]
