# Supplementary material for: Actin-regulated Siglec-1 nanoclustering influences HIV-1 capture and virus-containing compartment formation in dendritic cells
Source: eLife. 2023 Mar 20;12:e78836. doi: 10.7554/eLife.78836 (PMC10065798; doi:10.7554/eLife.78836)

Uncropped gel image of Figure 6-figure supplement 1E

p-Cofilin & actin
(1st blot from top)

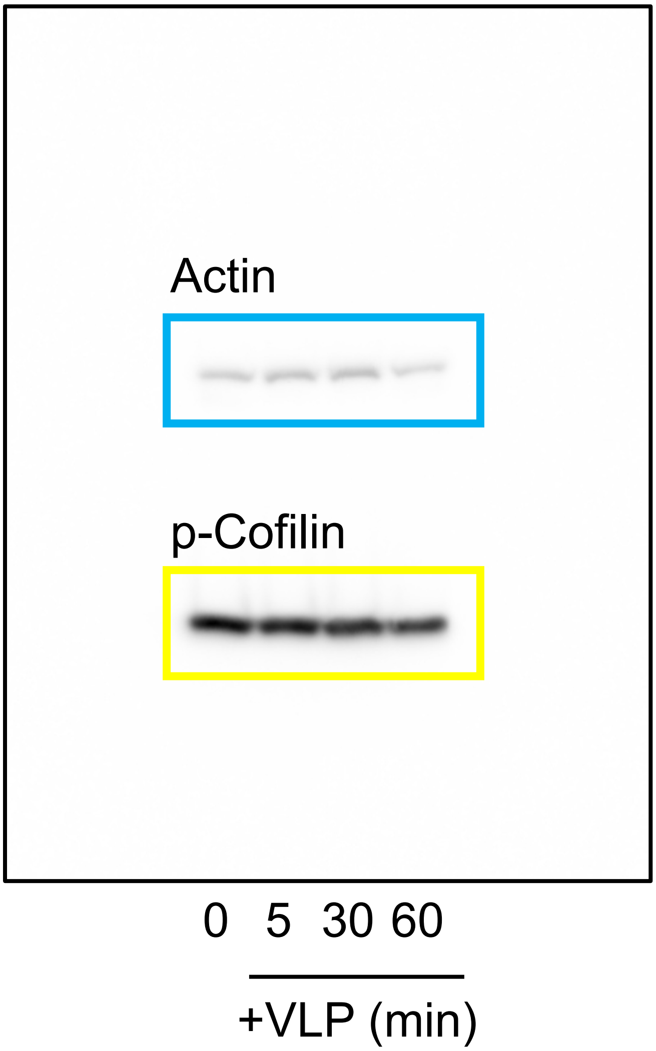

Cofilin & actin
(3rd blot from top)

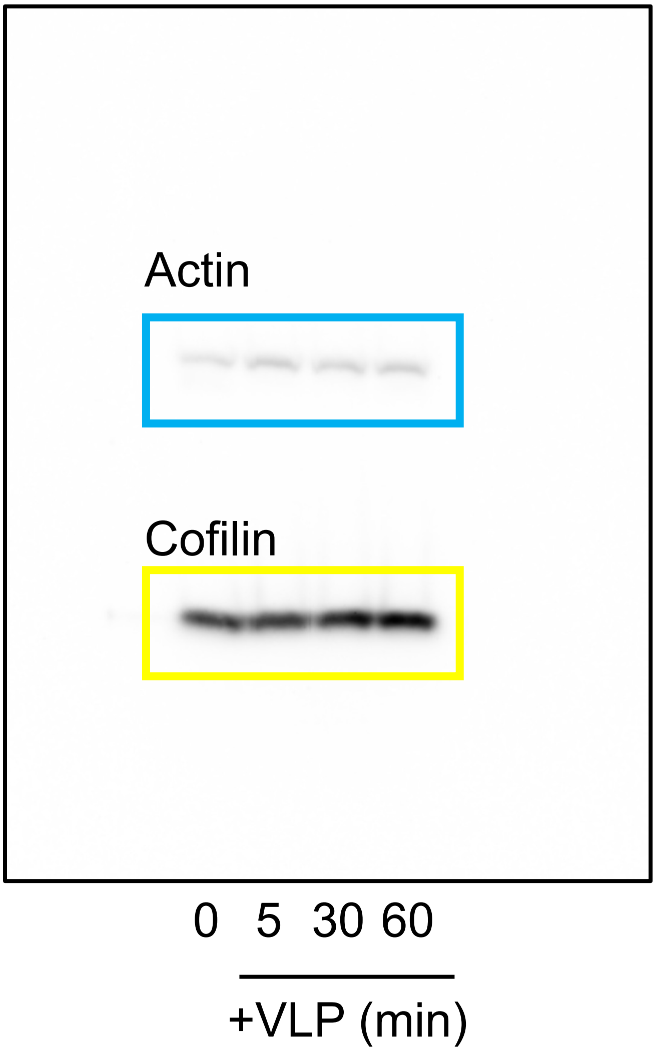

Same + picture of membranes with MW marker

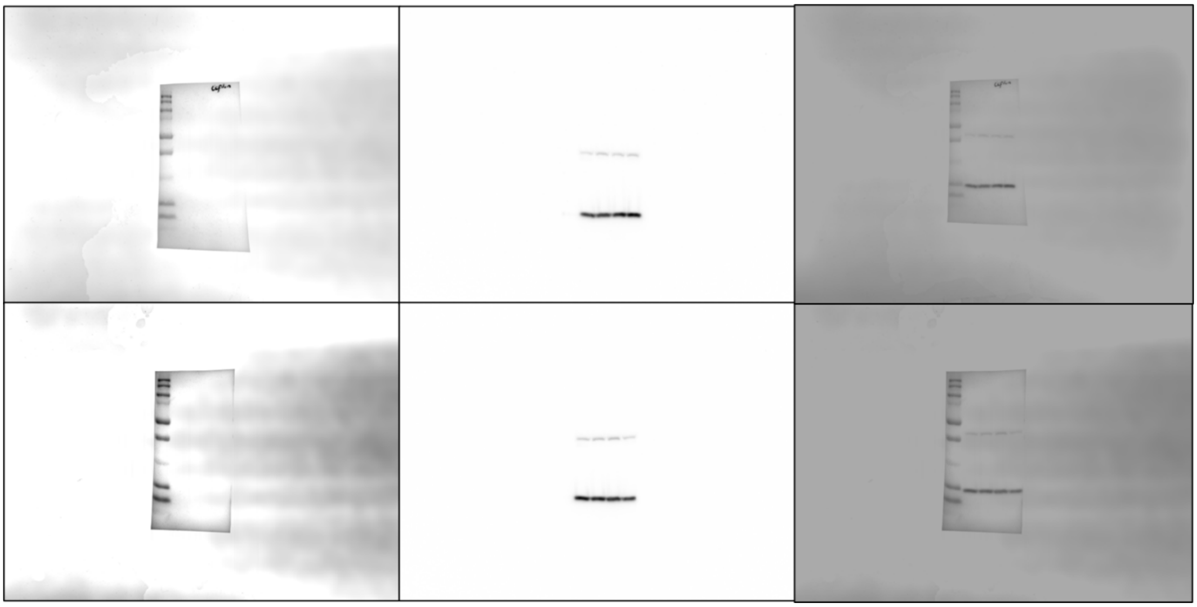

Supplement: Figure 6—figure supplement 1—source data 2. [file elife-78836-fig6-figsupp1-data2.zip › Figure 6-figure supplement 1-source data 2.pdf]
