# Supplementary material for: Actin-regulated Siglec-1 nanoclustering influences HIV-1 capture and virus-containing compartment formation in dendritic cells
Source: eLife. 2023 Mar 20;12:e78836. doi: 10.7554/eLife.78836 (PMC10065798; doi:10.7554/eLife.78836)
Supplement: MDAR checklist [file elife-78836-mdarchecklist1.docx]

**Materials Design Analysis Reporting (MDAR)**

**Checklist for Authors**

The [MDAR framework](https://osf.io/xfpn4/) establishes a minimum set of requirements in transparent reporting mainly applicable to studies in the life sciences.

*eLife* asks authors to **provide detailed information within their article** to facilitate the interpretation and replication of their work. Authors can also upload supporting materials to comply with relevant reporting guidelines for health-related research (see [EQUATOR Network](http://www.equator-network.org/%20)), life science research (see the [BioSharing Information Resource](http://biosharing.org/)), or animal research (see the [ARRIVE Guidelines](http://www.plosbiology.org/article/info:doi/10.1371/journal.pbio.1000412) and the [STRANGE Framework](https://doi.org/10.1038/d41586-020-01751-5); for details, see *eLife*’s [Journal Policies](https://reviewer.elifesciences.org/author-guide/journal-policies)). Where applicable, authors should refer to any relevant reporting standards materials in this form.

For all that apply, please note **where in the article** the information is provided. Please note that we also collect information about data availability and ethics in the submission form.

**Materials:**

| **Newly created materials** | **Indicate where provided: section/figure legend** | **N/A** |
| --- | --- | --- |
| The manuscript includes a dedicated "materials availability statement" providing transparent disclosure about availability of newly created materials including details on how materials can be accessed and describing any restrictions on access. |  | x |
|  |  |  |
| **Antibodies** | **Indicate where provided: section/figure legend** | **N/A** |
| For commercial reagents, provide supplier name, catalogue number and [RRID](https://scicrunch.org/resources), if available. | 1.Mouse monoclonal anti Siglec-1 (Hsn 7D20): Abcam (ab199401)  2.Rabbit polyclonal anti-cofilin: Abcam (Ab42824)  3. Rabbit polyclonal anti-phosphocofilin (phospho S3): Abcam (ab12866)  4. Rabbit antibodies anti phospho-Ezrin (Thr567)/Radixin (Thr564)/Moesin (Thr558) clone 48G2: Cell Signalling (3142)  5. Rabbit polyclonal Phospho-Myosin Light Chain 2 (Thr18/Ser19): Cell signalling (3674)  6. Anti-Mouse IgG F(ab) ATTO488: Hypermol (2112-250UG)  7. Cy3 Fab Fragment Donkey Anti- Rabbit: jacson Immunoresearch (711-167-003)  8. Actin Spirochrome Kit: (CY-SC001) Cytoskeleton  9. Rho Inhibitor I ADP ribosylation of Rho Asn-41: (CT04) Cytoskeleton  10. Rho Activator II  Deamidation of Rho Gln-63: (CN03) Cytoskeleton  11. Ezrin Inhibitor, NSC668394: Calbiochem (341216)  12. SMIFH2 formin inhibitor: S4826 Sigma  13. CK-666: SML0006 Sigma  14. Cytochalasin D: C8273 Sigma  15. ROCK inhibitor Y-27632: SCM075 Merck millipore. |  |
|  |  |  |
| **DNA and RNA sequences** | **Indicate where provided: section/figure legend** | **N/A** |
| Short novel DNA or RNA including primers, probes: Sequences should be included or deposited in a public repository. |  | x |
|  |  |  |
| **Cell materials** | **Indicate where provided: section/figure legend** | **N/A** |
| Cell lines: Provide species information, strain. Provide accession number in repository OR supplier name, catalog number, clone number, OR RRID. |  | x |
| Primary cultures: Provide species, strain, sex of origin, genetic modification status. | Peripheral blood mononuclear cells (PBMC) were |  |
|  |  |  |
| **Experimental animals** | **Indicate where provided: section/figure legend** | **N/A** |
| Laboratory animals or Model organisms: Provide species, strain, sex, age, genetic modification status. Provide accession number in repository OR supplier name, catalog number, clone number, OR RRID. |  | x |
| Animal observed in or captured from the field: Provide species, sex, and age where possible. |  | x |
|  |  |  |
| **Plants and microbes** | **Indicate where provided: section/figure legend** | **N/A** |
| Plants: provide species and strain, ecotype and cultivar where relevant, unique accession number if available, and source (including location for collected wild specimens). |  | x |
| Microbes: provide species and strain, unique accession number if available, and source. |  | x |
|  |  |  |
| **Human research participants** | **Indicate where provided: section/figure legend) or state if these demographics were not collected** | **N/A** |
| If collected and within the bounds of privacy constraints report on age, sex, gender and ethnicity for all study participants. |  | **x** |

**Design:**

| **Study protocol** | **Indicate where provided: section/figure legend** | **N/A** |
| --- | --- | --- |
| If the study protocol has been pre-registered, provide DOI. For clinical trials, provide the trial registration number OR cite DOI. |  | x |
|  |  |  |
| **Laboratory protocol** | **Indicate where provided: section/figure legend** | **N/A** |
| Provide DOI OR other citation details if detailed step-by-step protocols are available. |  | x |
|  |  |  |
| **Experimental study design (statistics details) *** | | |
| **For in vivo studies: State whether and how the following have been done** | **Indicate where provided: section/figure legend. If it could have been done, but was not, write “not done”** | **N/A** |
| Sample size determination |  | x |
| Randomisation |  | **x** |
| Blinding |  | x |
| Inclusion/exclusion criteria |  | x |
|  |  |  |
| **Sample definition and in-laboratory replication** | **Indicate where provided: section/figure legend** | **N/A** |
| State number of times the experiment was replicated in the laboratory. | For each experiment sample size, number of independent experiments and statistical results are detailed in the statistics section as well as in the Figure legends of the paper. |  |
| Define whether data describe technical or biological replicates. | We consider biological replicates experiments in which cell samples were obtained from different healthy donors of PBMCs. Technical replicates will refer to the number of measures (i.e.: number of cells analyzed for one experiment) performed from the biological samples of one PBMC donor. |  |
|  |  |  |
| **Ethics** | **Indicate where provided: section/submission form** | **N/A** |
| Studies involving human participants: State details of authority granting ethics approval (IRB or equivalent committee(s), provide reference number for approval. |  | x |
| Studies involving experimental animals: State details of authority granting ethics approval (IRB or equivalent committee(s), provide reference number for approval. |  | x |
| Studies involving specimen and field samples: State if relevant permits obtained, provide details of authority approving study; if none were required, explain why. |  | x |
|  |  |  |
| **Dual Use Research of Concern (DURC)** | **Indicate where provided: section/submission form** | **N/A** |
| If study is subject to dual use research of concern regulations, state the authority granting approval and reference number for the regulatory approval. |  | x |

**Analysis:**

| **Attrition** | **Indicate where provided: section/figure legend** | **N/A** |
| --- | --- | --- |
| Describe whether exclusion criteria were pre-established. Report if sample or data points were omitted from analysis. If yes, report if this was due to attrition or intentional exclusion and provide justification. |  | x |
|  |  |  |
| **Statistics** | **Indicate where provided: section/figure legend** | **N/A** |
| Describe statistical tests used and justify choice of tests. | All the data corresponding to statistical tests and sample size is included in the figure legends of the corresponding figures. A summary of all the statistical analysis used is found in the materials and methods section with the header “statistical analysis”  **Figure 1B:** Two way ANOVA; matched values are spread in rows (number of molecules per spot), in the columns we have cell type (immature DC vs. mature DC). N=4 experiments (4 source donors of PBMCs). In each individual experiment we calculated the average frequency of the number of molecules per spot of a minimum of 9 cells per condition (iDC vs. mDC).  We have a significant source of variation depending on the interaction between the row (number of molecules per spot) and the column (iDC vs. mDC) factors: 19.47% of total variation. P value = 0.0002  The significance presented between bars corresponds to a Bonferroni test in which we compare the mean value of number of molecules per spot frequency (i.e. 1, 2, 3 more than 4) in iDC vs. mDC.  For 1 molecules per spot (iDC vs. mDC): Mean diff = 0.351; 95% CI = 0.1733 to 0.5295; p=0.0003  For 2 molecules per spot (iDC vs. mDC): Mean diff = -0.2063 1; 95% CI = -0,3843 to -0,02826; p=0.021  **Figure 1C.** Paired T-test comparing the mean values of siglec-1 spot sizes (iDC vs. mDC). N=4 experiments. Each experiment shows the mean value of a minimum of 9 cells per condition analyzed.  Two-tailed P value = 0.0019  **Figure 1D.** Paired T-test comparing the mean values of the nearest neighbor distance (NND) between siglec-1 spots (iDC vs. mDC). N=4 experiments. Each experiment shows the mean value of a minimum of 9 cells per condition analyzed.  Two-tailed P value = 0.0350  **Figure 1G.** Ratio Paired T-test comparing the fraction of siglec-1 immobile molecules. N=3 experiments. For each experiment we measured the average immobile fraction of at least 3 cells per experiment (in total 16 iDC cells and 15 mDC cells). In mDC one outliner was removed from the third experiment (value was > mean + 2 times SD).  Two-tailed P value = 0.0036  **Figure 1H**: Two way ANOVA; matched values are spread in rows (MSS slope <0.2, between 0.2 and 0.5; > 0.5), in the columns we have cell type (immature DC vs. mature DC). N=3 experiments. For each cell analyzed we calculated the frequency of MSS slopes in the ranges described above. For each experiment we calculated the average frequencies of a minimum of 3 cells per condition (iDC vs. mDC). We analyzed a total of 16 iDC and 18 mDC. One outliner was removed from mDC (value was > mean + 2 times SD).  We have a significant source of variation depending on the interaction between the row factor (MSS slope <0.2, between 0.2 and 0.5; > 0.5) and the column factor (iDC vs. mDC) factors: 11.41% of total variation. P value = 0.0004  The significance presented between bars corresponds to a Bonferroni test in which we compare the mean value of number of each MSS slope classification in iDC vs. mDC.  For MSS slope < 0.2. Mean diff (iDC vs. mDC)= 0.1228; 95% CI = 0.05603 to 0.1896. P value 0.0028  For MSS slope 0.2 – 0.5 (iDC vs. mDC) Mean diff = -0.1239; 95% CI = -0,1907 to -0,05707. P value 0.0027  **Figure 2F.** Ratio paired t-test of average voronoi areas between contiguous siglec-1 spots per cell in control and CK666 treated cells.  (N=2; p = 0.92, two-tailed, non-significant). For the first experiment we counted 16 control cells (one outliner removed; value > 2 times S.D) and 17 cells treated with CK666. For the second experiment we counted 10 control and 10 CK666 treated cells.  For each individual experiment we assessed the significance with a non- parametric Mann Whitney test.  Experiment 1: p=0.4559 two-tailed (non significant)  Experiment 2: p=0.852 two-tailed (non significant)  **Figure 2G**. Ratio paired t-test of average voronoi areas between contiguous siglec-1 spots per cell in control and SMIFH2 treated cells. (N=3); For the first experiment we counted 19 control cells and 18 cells treated with SMIFH2. For the second experiment we counted 17 control and 19 SMIFH2 treated cells. For the third experiment we counted 10 control and 10 SMIFH2 treated cells.  For each individual experiment we assessed the significance with a non- parametric Mann Whitney test.  Experiment 1: p = 0.0269, two-tailed  Experiment 2: p=0.0181 two-tailed  Experiment 3: p=0.3150 two-tailed (non significant)  With the average of biological replicates (N=3 donors) we performed a ratio paired T test.  Two-tailed P value = 0.0298  **Figure 2H:** Two way ANOVA matched values are spread in rows (number of molecules per spot), in the columns we have cell type (mDC vs. mDC+ ck666). N=2 experiments. Experiment 1: 9 control cells vs 10 CK666; Experiment 2: 10 control cells vs 10 CK666. We calculated the frequency of molecules per spots (i.e 1, 2, 3, >4) pulling together all the clusters measured in all the cells analyzed (control vs. CK666) for each experiment.  We do not have a significant source of variation depending on the interaction between the row (number of molecules per spot) and the column (mDC vs. mDC + CK666) factors: 1.596% of total variation. P value = 0.4902  Bonferroni test in which we compared the mean value of number of molecules per spot frequency (i.e. 1, 2, 3 more than 4) in mDC vs. mDC + CK666 did not give any significant value.  **Figure 2I:** Two way ANOVA matched values are spread in rows (number of molecules per spot), in the columns we have cell type (mDC vs. mDC+ SMIFH2). N=3 experiments. Experiment 1: 9 control cells vs 10 SMIFH2; Experiment 2: 10 control cells vs 13 SMIFH2; Experiment 3: 18 control cells vs 18 SMIFH2.  We have a significant source of variation depending on the interaction between the row (number of molecules per spot) and the column (mDC vs. mDC + SMIFH2) factors: 12.29% of total variation. P value = 0.0002  The significance presented between bars corresponds to a Bonferroni test in which we compare the mean value of number of molecules per spot frequency (i.e. 1, 2, 3 more than 4) in mDC vs. mDC + SMIFH2.  For 1 molecules per spot (mDC vs. mDC + SMIFH2): Mean diff = -0.2098; 95%; CI = -0,3027 to -0,1169; p=0.0004  For 2 molecules per spot (mDC vs. mDC + SMIFH2): Mean diff = 0.1014; 95%; CI = 0,008467 to 0,1943; p=0.0324.  **Figure 2K.** Ratio Paired T-test comparing the fraction of siglec-1 immobile molecules. N=3 experiments. For the first experiment we measured the average immobile fraction of 7 control cells and 9 cells treated with SMIFH2, for the second experiment 7 control and 6 cell treated with SMIFH2, for the third experiment 7 control and 8 SMIFH2 (in total 21 mDC cells and 23 mDC + SMIFH2 cells).  Two-tailed P value =0.039  **Figure 2L:** Two way ANOVA; matched values are spread in rows (MSS slope <0.2, between 0.2 and 0.5; > 0.5), in the columns we have cell type (mature DC vs. mature DC + SMIFH2). N=3 experiments (3 source donors of PBMCs). For each cell analyzed we calculated the frequency of MSS slopes in the ranges described above. Experiment 1 = 7 control and 6 SMIFH2 tretaed cells; Experiment 2 = 7 control and 8 SMIFH2 cells; Experiment 3 = 8 control and 8 SMIFH2 (mDC= 21 cells vs. mDC + SMIFH2=22 cells).  We have a significant source of variation depending on the interaction between the row factor (MSS slope <0.2, between 0.2 and 0.5; > 0.5) and the column factor (mDC vs. mDC + SMIFH2) factors: 1.18% of total variation. P value = 0.0187  The significance presented between bars corresponds to a Bonferroni test in which we compare the mean value of number of each MSS slope classification in mDC and mDC + SMIFH2.  For MSS slope < 0.2. Mean diff (mDC vs. mDC + SMIFH2) = 0.06619; 95%; CI = 0,006650 to 0,1257; P value = 0.0238  **Figure 3B:** Two way ANOVA in which matched values are spread in rows (distance from the cell base). In the columns we have relative value of siglec-1 intensity in mDC vs iDC. N=2. Experiment 1: 15 iDC and 10 mDC. Experiment 2 = 10 iDC and 10mDC. The graphs show the mean + S.E.M of siglec-1 relative intensity from the cell base.  We have a significant source of variation depending on the column factor (iDC vs. mDC): 32,91% of total variation; p<0,0001. The stars in the graph refer to this parameter.  We have a significant source of variation depending on the interaction between the row factor (distance from the cell base) and the column factor (iDC vs mDC) factors: 17,11% of total variation; p= 0,047  We have a significant source of variation depending on the row factor (distance from the cell base): 56,91% of total variation; p= 0,0269.  **Figure 3C:** Two way ANOVA in which matched values are spread in rows (distance from the cell base). In the columns we have relative value of pERM intensity in mDC vs iDC. N=2. Experiment 1: 15 iDC and 10 mDC. Experiment 2 = 10 iDC and 10mDC. The graphs show the mean + S.E.M of pERM relative intensity from the cell base.  We have a significant source of variation depending on the column factor (iDC vs. mDC): 6,365% of total variation; p=0,0148. The stars in the graph refer to this parameter.  We have a significant source of variation depending on the interaction between the row factor (distance from the cell base) and the column factor (iDC vs mDC) factors: 16,67% of total variation; p= 0,047  We have a significant source of variation depending on the row factor (distance from the cell base): 56,91% of total variation; p= 0,0269  **Figure 3F:** Two way ANOVA in which matched values are spread in rows (distance from the cell base). In the columns we have relative value of siglec-1 intensity in mDC vs mDC + CT04. N=2. Experiment 1 11 mDC and 9 mDC + CT04. Experiment 2 = 10 mDC and 7 mDC+ CTC04. The graphs show the mean + S.E.M of siglec-1 relative intensity from the cell base.  We have a significant source of variation depending on the column factor (mDC vs. mDC + CT04): 16.4 % of total variation; p= 0,0238. The stars in the graph refer to this parameter.  **Figure 3G:** Two way ANOVA matched values are spread in rows (distance from the cell base) in the columns we have relative value of siglec-1 intensity in mDC vs mDC + Y27623. N=2. Experiment 1: 10 mDC and 12 mDC + Y27623. Experiment 2 = 15 mDC and 21 mDC+ Y27623. The graphs show the mean + S.E.M of siglec-1 relative siglec-1 intensity from the cell base.  We have a significant source of variation depending on the column factor (mDC vs. mDC + Y27623): 26.67 % of total variation; p= 0.0009. The stars in the graph refer to this parameter.  We have a significant source of variation depending on the interaction between the row factor (distance from the cell base): 26,35% of total variation; p= 0,0008  Bonferroni test in which we compare the relative siglec-1 intensity at different distances from the cell base in mDC vs. mDC + CT04 gave the following significant differences:  Relative siglec-1 intensity at 0 to 1.5 µm from the base (mDC vs. mDC + Y27623). Mean diff = 1,562  ; 95% CI = 0,1231 to 3,002; P= 0,032.  **Figure 3H.** Non parametric Mann Whitney test. We show the mean + SD of the average voronoi areas between contiguous Siglec-1 spots in control mDC, and cells treated with CT04. One representative experiment out of 2. Experiment 1 = 17 mDC and 16 mDC + CT04. Experiment 2 = 15 mDC and 13 mDC + CT04.  Experiment 1: P = 0.0002 two tailed  Shown in paper  Experiment 2: P = 0.0026 two tailed  **Figure 3I.** Non parametric Mann Whitney test. We show the mean + SD of the average voronoi areas between contiguous Siglec-1 spots in control mDC, and cells treated with Y27623. One representative experiment out of 2. Experiment 1 = 12 mDC and 14 mDC + Y27623. Experiment 2 = 14 mDC and 16 mDC + Y27623.  Experiment 1: P = 0.0127 two tailed  Experiment 2: P = 0.0001 two tailed Shown in paper  **Figure 3J:** Two way ANOVA; matched values are spread in rows (number of molecules per spot), in the columns we have cell type (control mDC vs. mDC + CT04). N=2 experiments. In each individual experiment we calculated the average frequency of the number of molecules per spot. Experiment 1: 10 mDC and 11 mDC + CT04. Experiment 2: 15 mDC and 13 mDC + CT04.  We have a significant source of variation depending on the interaction between the row (number of molecules per spot) and the column (mDC vs. mDC + CT04) factors: 9.026 % of total variation. P value = 0.0045  The significance presented between bars corresponds to a Bonferroni test in which we compare the mean value of number of molecules per spot frequency (i.e. 1, 2, 3 more than 4) in mDC vs. mDC + CT04.  For 1 molecules per spot (mDC vs. mDC + CT04): Mean diff = -0.1961; 95% CI = -0,3083 to -0,08397; p=0.0068  **Figure 3K:** Two way ANOVA; matched values are spread in rows (number of molecules per spot), in the columns we have cell type (control mDC vs. mDC + Y127623). N=2 experiments. In each individual experiment we calculated the average frequency of the number of molecules per spot. Experiment 1: 12 mDC and 9 mDC + Y27623. Experiment 2: 12 mDC and 16 mDC + Y127623.  We have a significant source of variation depending on the interaction between the row (number of molecules per spot) and the column (mDC vs. mDC + CT04) factors: 14.51 % of total variation. P value = 0.0057  The significance presented between bars corresponds to a Bonferroni test in which we compare the mean value of number of molecules per spot frequency (i.e. 1, 2, 3 more than 4) in mDC vs. mDC + Y127623.  For 1 molecules per spot (mDC vs. mDC + Y127623): Mean diff = -0,2601; 95% CI = -0,4186 to -0,1017; p=0.0084.  Figure 4B. Non parametric Mann Whitney test. We show the mean + SD of VLP-GagCherry in control mDC, and cells treated with SMIFH2. One representative experiment out of 2. Experiment 1 = 14 mDC and 14 mDC + SMIFH2. Experiment 2 = 15 mDC and 15 mDC + SMIFH2.  Experiment 1: P = 0.0006 two tailed. Shown in paper  Experiment 2: P = 0.0086 two tailed  **Figure 4C.** Non parametric Mann Whitney test. We show the mean + SD of siglec-1 in control mDC, and cells treated with SMIFH2. One representative experiment out of 2. Experiment 1 = 14 mDC and 14 mDC + 15 SMIFH. Experiment 2 = 14 mDC and 16 mDC + SMIFH2.  Experiment 1: P = 0.5907 two tailed. Not significant. Shown in paper  Experiment 2: P = 0.1417 two tailed. Not significant  **Figure 4D.** Non parametric Mann Whitney test. We show the average siglec-1 molecules per spot per cell in control mDC and cells treated with SMIFH2 at steady-state and after a 5 minutes pulse with VLPGag-Cherry. For the steady-state we performed 4 experiments (Experiment 1= 9 control and 10 SMIFH2; Experiment 2 = 17 control + 17 SMIFH2; Experiment 3 = 14 control + 10 SMIFH2 ). For the cells pulsed with VLPGag-Cherry we performed 2 experiments (Experiment 1 = 10 control + 8 SMIFH2; Experiment 2 = 10 control + 8 SMIFH2). In the cells pulsed with VLPGag-Cherry we divided the population of siglec-1 receptors according to their colocalization with VLPs.  Set of experiments in the absence of VLPs:  Experiment 1: P = 0.0821 two tailed.  Experiment 2: P <0,0001 two tailed.  Experiment 3: P = 0,0358 two tailed  Shown in paper  Set of experiments in the absence of VLPs:  Experiment 1 (5 minutes) : P = 0,0343 two tailed  Shown in paper  Experiment 2 (5 minutes) : P = 0,0592 two tailed.  Experiment 1 (30 minutes) : P = 0,2031 two tailed  Shown in paper  Experiment 2 (30 minutes) : P = 0,2359 two tailed.  Experiment 1 (60 minutes) : P = 0,4082 two tailed  Shown in paper  Experiment 2 (60 minutes) : P = 0,4807two tailed.  **Figure 4F.** Kruskal-Wallis test. We show LUV intensity in mDC after 5 min of LUV capture. Results from one experiment in which we measured 15 cells pulsed with 0.25% GM1 LUVs, 15 cells pulsed with 0.5% GM1 LUVs and 14 cells pulsed with 4% GM1.  The significance presented between bars corresponds to a Kruskal-Wallis test with a Dunn's multiple comparisons test.  0.25% vs. 0.5%; Mean rank difference = 6,333; Adjusted p-value = 0,5308  0.25% vs. 4%; Mean rank difference = -18,83; Adjusted p-value = 0,0002  0.5% vs. 4%; ean rank difference = -25,17; Adjusted p-value = <0,0001  Figure 4G. Kruskal-Wallis test. We show the average siglec-1 cluster size per cell colocalizing with GM-1 LUVs in mDC after 5 min capture. Results from one experiment in which we measured 13 cells pulsed with 0.25% GM1 LUVs, 12 cells pulsed with 0.5% GM1 LUVs and 13 cells pulsed with 4% GM1.  The significance presented between bars corresponds to a Kruskal-Wallis test with a Dunn's multiple comparisons test.  0.25% vs. 0.5%; Mean rank difference = 0,2756; Adjusted p-value = >0,9999  0.25% vs. 4%; Mean rank difference = 12; Adjusted p-value = 0,0177  0.5% vs. 4%; Mean rank difference = 11,72; Adjusted p-value = 0,0252  Figure 4H: Two way ANOVA; matched values are spread in rows (number of molecules per spot), in the columns we have different GM-1 concentrations in LUVs (0.25% vs. 0.5% vs. 4%). Results from one experiment in which we measured 12 cells pulsed with 0.25% GM-1, 13 cells pulsed with 0.5% GM-1 and 13 cells pulsed with 4% GM-1.  We have a significant source of variation depending on the interaction between the row (number of molecules per spot) and the column (GM-1 concentration) factors: 12,17% of total variation. P value <0,0001  The significance presented between bars corresponds to a Bonferroni test in which we compare the mean value of number of molecules per spot frequency (i.e. 1, 2, 3 more than 4) in mDCs treated with different concentrations of GM-1.  For 2 molecules per spot :  0.25 vs 4: Mean diff = -0,2254 ; 95% CI = -0,3753 to -0,07546; p=0,0011  0.5 vs 4: Mean diff =-0,2042 ; 95% CI = -0,3572 to -0,05123; p=0,0046  For 3 molecules per spot :  0.25 vs 4: Mean diff = -0,1783; 95% CI = -0,3282 to -0,02836; p= 0,0137  For >4 molecules per spot :  0.25 vs 4: Mean diff = 0,3650; 95% CI = 0,2151 to 0,5149; p <0,0001  0.5 vs 4: Mean diff =0,2572 ; 95% CI = 0,1042 to 0,4102; p=0,0002  **Figure 4J.** Kruskal-Wallis test. We show the average siglec-1 density of molecules at different radius from colocalizing spots in mDC pulsed for 5 minutes with LUVs (0.25%, 0.5% and 4% GM-1). Results from one experiment in which we measured 13 cells pulsed with 0.25% GM1 LUVs, 11 cells pulsed with 0.5% GM1 LUVs and 12 cells pulsed with 4% GM1.  The significance presented between bars corresponds to a Kruskal-Wallis test with a Dunn's multiple comparisons test.  For the cells pulsed with 0.25% GM-1:  0.6um vs. 1.2um: P= 0,0150; 0.6um vs. 1.2um: P= <0,0001; 1.2um vs. 3 um: P= 0,0725;  For the cells pulsed with 0.5% GM-1:  0.6um vs. 1.2um: P= 0,0093; 0.6um vs. 1.2um: P= <0,0001; 1.2um vs. 3 um: P= 0,0090;  For the cells pulsed with 0.4% GM-1:  0.6um vs. 1.2um: P= <0,0001; 0.6um vs. 1.2um: P= <0,0001; 1.2um vs. 3 um: P= <0,0001;  **Figure 5B:** Two way ANOVA; in rows we have number of molecules per spot, in the columns we have time (5, 30 and 60 minutes). Results from one experiment in which we analysed 10 cells at 5 minutes, 10 cells at 30 minutes and 10 cells at 60 minutes.  We have a significant source of variation depending on the interaction between the row (number of molecules per spot) and the column (5 vs. 30 vs. 60 minutes) factors: 16.14 % of total variation. P value <0,0001  The significance presented between bars corresponds to a Bonferroni test in which we compare the mean value of number of molecules per spot frequency (i.e. 1, 2, 3 more than 4) at different times of incubation with VLPs.  For 2 molecules per spot:  5 minutes vs 60 minutes : Mean diff = 0,2184; 95% CI = 0,03982 to 0,3971; p= 0,0109  30 minutes vs 60 minutes : Mean diff =0,3118 ; 95% CI = 0,1331 to 0,4904; p= 0,0001  For 4 molecules per spot:  30 minutes vs 60 minutes : Mean diff = -0,2300; 95% CI = -0,4086 to -0,05138; p= 0,0067  **Figure 5C:** Two way ANOVA; in rows we have number of molecules per spot, in the columns we have time (5, 30 and 60 minutes). Results from one experiment in which we analysed 9 cells at 5 minutes, 10 cells at 30 minutes and 10 cells at 60 minutes.  We have a significant source of variation depending on the interaction between the row (number of molecules per spot) and the column (5 vs. 30 vs. 60 minutes) factors: 16.14 % of total variation. P value <0,0001  The significance presented between bars corresponds to a Bonferroni test in which we compare the mean value of number of molecules per spot frequency (i.e. 1, 2, 3 more than 4) at different times of incubation with VLPs.  For 3 molecules per spot:  5 minutes vs 30 minutes : Mean diff = 0,2178 ; 95% CI = 0,06031 to 0,3753; p= 0,0039  30 minutes vs 60 minutes : Mean diff = 0,1996 ; 95% CI = 0,04216 to 0,3571; p= 0,0090  For 4 molecules per spot:  5 minutes vs 60 minutes : Mean diff = -0,3392; 95% CI = -0,4967 to -0,1817; p <0,0001  5 minutes vs 60 minutes : Mean diff = -0,3097; 95% CI =-0,4672 to -0,1523; p <0,0001  **Figure 5D:** Two way ANOVA; in rows we have average siglec-1 molecules per spot per cell normalized to the average value after 5 minutes incubation VLPs, in the columns we have cell type (iDC vs. mDC). Results from one experiment in which we analyzed 10 iDC and 10 mDC cells at 30 minutes and 60 minutes of incubation with VLPs.  We have a significant source of variation depending on the row factor (molecules per spot at different times): 20,18 % of total variation, p = 0,0001.  We have a significant source of variation depending on the column factor factor (iDC vs. mDC): 37.66 % of total variation, p < 0,0001.  The significance presented between bars corresponds to a Bonferroni test:  iDC vs. mDC at 30 minutes: Mean diff = -0,4953; 95% CI = -0,7174 to -0,2732 ; p <0,0001  iDC vs. mDC at 60 minutes: Mean diff = -0,2904; 95% CI = -0,5125 to -0,06829 ; p = 0,0084  30min vs 60min (in iDC): Mean diff = -0,3900; 95% CI = -0,6121 to -0,1679; p = 0,0004  30min vs 60min (in mDC): Mean diff =-0,1851; 95% CI=-0,4072 to 0,03701; p=0,1182  **Figure 5E.** Kruskal-Wallis test. We show the distribution of VLP intensity from the center of mass after different times of incubation (5, 30 and 60 minutes) in iDC. For the statistics shown in the legend we used the distance from the center of mass at which we recover 50% of the total intensity. Results from one experiment in which we analyzed 10 cells at 5 minutes, 8 cells at 30 minutes and 10 cells at 60 minutes.  The significance presented between bars corresponds to a Kruskal-Wallis test with a Dunn's multiple comparisons test.  iDC 60min vs. iDC 30min: Mean rank difference = -6,000; Adjusted p-value = 0,3453.  iDC 60min vs. iDC 5min: Mean rank difference = -4,933; Adjusted p-value = 0,6219.  iDC 30min vs. iDC 5min: Mean rank difference = 1,067; Adjusted p-value >0,9999  **Figure 5F.** Kruskal-Wallis test. We show the distribution of VLP intensity from the center of mass after different times of incubation (5, 30 and 60 minutes) in mDC. For the statistics shown in the legend we used the distance from the center of mass at which we recover 50% of the total intensity. Results from one experiment in which we analyzed 9 cells at 5 minutes, 10 cells at 30 minutes and 10 cells at 60 minutes.  The significance presented between bars corresponds to a Kruskal-Wallis test with a Dunn's multiple comparisons test.  mDC 60min vs. mDC 30min: Mean rank difference = -6,078; Adjusted p-value = 0,3233.  mDC 60min vs. mDC 5min: Mean rank difference = -15,89; Adjusted p-value = 0,0001  mDC 30min vs. mDC 5min: Mean rank difference = -9,811; Adjusted p-value = 0,0283  **Figure 5G:** Two way ANOVA; in rows we have average siglec-1 molecules per spot per cell normalized to the average value after 5 minutes incubation LUVs, in the columns we have different GM-1 concentrations (4% vs. 0.5%. Results from one experiment in which we analyzed 14 cells incubated with 4% GM-1 at 30 minutes, 15 cells incubated with 4% GM-1 at 60 minutes, 16 cells incubated with 0.5% GM-1 at 30 minutes, 13 cells incubated with 0.5% GM-1 at 60 minutes,  We have a significant source of variation depending on the row factor (molecules per spot at different times): 11,33 % of total variation, p = 0,0031.  We have a significant source of variation depending on the column factor (GM-1 concentration): 19,85 % of total variation, p = 0,0001  The significance presented between bars corresponds to a Bonferroni test:  30 minutes vs 60 minutes (4% GM-1 LUVs) : Mean diff =-0,1284 ; 95% CI = -0,3703 to 0,1135; p = 0,4526  30 minutes vs 60 minutes (0.5 % GM-1 LUVs): Mean diff = -0,3318 ; 95% CI = -0,5748 to -0,08869 ; p = 0,0054  4% GM-1 vs 0.5% GM-1 (30 minutes) : Mean diff =0,4063 ; 95% CI = 0,1680 to 0,6445 ; p = 0,0005  4% GM-1 vs 0.5% GM-1 (60 minutes) : Mean diff =0,2029 ; 95% CI =-0,04375 to 0,4496; p = 0,1264  **Figure 5H.** Kruskal-Wallis test. We show the distribution of VLP intensity from the center of mass after different times of incubation with 4% GM-1 LUVs (5, 30 and 60 minutes) in mDC. For the statistics shown in the legend we used the distance from the center of mass at which we recover 50% of the total intensity. Results from one experiment in which we analyzed 15 cells at 5 minutes, 7 cells at 30 minutes and 12 cells at 60 minutes.  The significance presented between bars corresponds to a Kruskal-Wallis test with a Dunn's multiple comparisons test.  mDC 60min vs. mDC 30min (4% GM-1 LUV): Mean rank difference = -0,2208 ; Adjusted p-value >0,9999  mDC 60min vs. mDC 5min (4% GM-1 LUV): Mean rank difference = -16,1 ; Adjusted p-value <0,0001  mDC 30min vs. mDC 5min (4% GM-1 LUV): Mean rank difference = -15,88 ; Adjusted p-value =0,001  **Figure 5I.** Kruskal-Wallis test. We show the distribution of VLP intensity from the center of mass after different times of incubation with 0.5 % GM-1 LUVs (5, 30 and 60 minutes) in mDC. For the statistics shown in the legend we used the distance from the center of mass at which we recover 50% of the total intensity. Results from one experiment in which we analyzed 14 cells at 5 minutes, 8 cells at 30 minutes and 13 cells at 60 minutes.  The significance presented between bars corresponds to a Kruskal-Wallis test with a Dunn's multiple comparisons test.  mDC 60min vs. mDC 30min (0.5% GM-1 LUV): Mean rank difference = -6,078 ; Adjusted p-value = 0,3233  mDC 60min vs. mDC 5min (0.5% GM-1 LUV): Mean rank difference = -15,89 ; Adjusted p-value = 0,0001  mDC 30min vs. mDC 5min (0.5% GM-1 LUV): Mean rank difference = -9,811 ; Adjusted p-value =0,0283  **Figure 5J.** Kruskal-Wallis test. We show the average distance per cell of non-colocalizing siglec-1 spots to siglec-1 colocalizing with VLP spots in mDC at different times of incubation with VLPs (5 minutes, 30 minutes and 60 minutes). Results from one experiment in which we analyzed 9 cells at 5 minutes, 10 cells at 30 minutes and 10 cells at 60 minutes.  The significance presented between bars corresponds to a Kruskal-Wallis test with a Dunn's multiple comparisons test.  mDC 5min vs. mDC 30min: Mean rank difference = 9,622 ; Adjusted p-value = 0,0417  mDC 5min vs. mDC 60min: Mean rank difference = 17,12 ; Adjusted p-value <0,0001  mDC 30min vs. mDC 60min: Mean rank difference = 7,5 ; Adjusted p-value = 0,1467  **Figure 5K:** Two way ANOVA; in rows we have time of incubation with LUVs, in the columns we have the concentrations of GM-1 (0.5% and 4%). The variable assessed is the average distance per cell of non-colocalizing siglec-1 dots with respect to colocalizing siglec -1 dots. Results from one experiment in which we analyzed for 4% GM-1 LUVs 12 cells at 5 minutes of incubation, 14 cells at 30 minutes of incubation and 15 cells at 60 minutes of incubation and for 0.5 % GM-1 LUVs 11 cells at 5 minutes of incubation, 16 cells at 30 minutes of incubation and 14 cells at 60 minutes of incubation  We have a significant source of variation depending on the interaction between the column factor (concentration of GM-1) and the row factor (time of incubation): 11,49 % of total variation, p <0,0001.  We have a significant source of variation depending on the row factor: 46,32 % of total variation, p < 0,0001.  The significance presented between bars corresponds to a Bonferroni test:  5 minutes vs 30 minutes (4% GM-1) : Mean diff = 3,07; 95% CI = 1,661 to 4,481; p <0,0001  5 minutes vs 60 minutes (4% GM-1): Mean diff = 4,422; 95% CI = 3,034 to 5,810; p <0,0001  30 minutes vs 60 minutes (4% GM-1): Mean diff = 1,351; 95% CI =0,01906 to 2,683; p = 0,0457  5 minutes vs 30 minutes (0.5% GM-1) : Mean diff =-0,6834; 95% CI = -2,087 to 0,7204; p = 0,7112  5 minutes vs 60 minutes (0.5% GM-1): Mean diff = 3,095; 95% CI = 1,651 to 4,539; p <0,0001  30 minutes vs 60 minutes (0.5% GM-1): Mean diff =3,779; 95% CI =2,467 to 5,090; p<0,0001  **Figure 6B:** Two way ANOVA; in rows we have distance from the cell base, in the columns we have time of incubation with VLPs (5 minutes, 30 minutes and 60 minutes). The variable assessed is the cell area depending on distance from the cell base and time of incubation with VLPs. For the 5 minutes time we plotted the average values of 4 independendent expetriments (experiment 1: 10 cells, experiment 2: 15 cells, experiment 3: 13 cells, experiment 4: 15 cells). For the 30 minutes time we plotted the average values of 4 independendent expetriments (experiment 1: 13 cells, experiment 2: 13 cells, experiment 3: 14 cells, experiment 4: 7 cells). For the 60 minutes time we plotted the average values of 3 independendent expetriments (experiment 1: 11cells, experiment 2: 9 cells, experiment 3: 17 cells).  The significance shown in the figure legend between the different times of incubation with VLPs corresponds to the percentage of variance due to the interaction between the column factor (time of incubation with VLPs) and the row factor (distance from the cell base).  5 minutes vs. 30 minutes: 23,83 % of total variation; p= 0,0009  5 minutes vs. 60 minutes: 21,63 % of total variation; p= 0,0065  30 minutes vs. 60 minutes: 0,8274 % of total variation; p= 0,9983  **Figure 6C:** Two way ANOVA; in rows we have distance from the cell base, in the columns we have LUVs with different concentrations of GM-1 (0.5% vs. 4%). The variable assessed is the cell area depending on distance from the cell base after 5 minutes of incubation with the LUVs. Results correspond to one experiment in which we measured 12 cells for 0.5% GM-1 LUVs and 15 cells for 4% GM-1 LUVs  The significance shown in the figure legend corresponds to the percentage of variance due to the interaction between the column factor (GM-1 concentration) and the row factor (distance from the cell base): 0,9918 % of total variation; p= 0,6133.  **Figure 6D**: Two way ANOVA; in rows we have distance from the cell base, in the columns we have LUVs with different concentrations of GM-1 (0.5% vs. 4%). The variable assessed is the cell area depending on distance from the cell base after 30 minutes of incubation with the LUVs. Results correspond to one experiment in which we measured 8 cells for 0.5% GM-1 LUVs and 7 cells for 4% GM-1 LUVs  The significance shown in the figure legend corresponds to the percentage of variance due to the interaction between the column factor (GM-1 concentration) and the row factor (distance from the cell base): 19,2 % of total variation; p= <0,0001  **Figure 6E:** Two way ANOVA; in rows we have distance from the cell base, in the columns we have LUVs with different concentrations of GM-1 (0.5% vs. 4%). The variable assessed is the cell area depending on distance from the cell base after 60 minutes of incubation with the LUVs. Results correspond to one experiment in which we measured 14 cells for 0.5% GM-1 LUVs and 12 cells for 4% GM-1 LUVs  The significance shown in the figure legend corresponds to the percentage of variance due to the interaction between the column factor (GM-1 concentration) and the row factor (distance from the cell base): 7,125% of total variation; p= 0,0197.  **Figure 6G.** Paired T-test comparing the mean values of pERM intensity in mDC at steady-state and at 60 minutes with incubation with VLPs. N=3 experiments. Experiment 1: 12 cell 0 minutes and 10 cells at 60 minutes. Experiment 2: 40 cells 0 minutes and 40 cells at 60 minutes. Experiment 3: 15 cells 0 minutes and 7 cells 60 minutes.  Two-tailed P value = 0,0162.  **Figure 6H:** Two way ANOVA; in rows we have time of incubation with VLPs, in the columns we have treatment (control mDC vs. mDC + CN03). The variable assessed is the mean intensity of pERM depending on time of incubation with VLPs and treatment. Results correspond to one experiment in which we measured 15 control cells and 9 mDC+CN03 cells in the absence of VLPs and 7 control cells and 15 mDC+CN03 cells after 1h incubation with VLPs.  We have a significant source of variance due to the interaction between the column factor (treatment) and the row factor (time of incubation with VLPs): 5,082 % of total variation; p= 0,0418.  We have a significant source of variance due to the interaction between the column factor (treatment): 20,14% of total variation; p= 0,0001  We have a significant source of variance due to the row factor (time of incubation with VLPs): 35,38 % of total variation; p <0,0001.  The significance shown in the graph correspond to a Bonferroni multiple comparison test:  Control mDC vs mDC + CN03 (no VLP) : Mean diff = -16,59; 95% CI =-23,42 to -9,749; p <0,0001  Control mDC vs mDC + CN03 (60 minutes VLP) : Mean diff = -7,469 ; 95% CI = -14,89 to -0,04711; p= 0,0483  **Figure 6I .** Non parametric Mann Whitney test. We show the distribution of VLP intensity from the center of mass after 60 minutes of incubation in mDC and cells treated with CN03. For the statistics shown in the legend we used the distance from the center of mass at which we recover 50% of the total intensity. Results from one experiment in which we analyzed 7 control cells and 12 cells treated with CN03.  Two-taile p-value= 0,0171  **Figure 6J:** Two way ANOVA; in rows we have distance from the cell base, in the columns we have treatement (control mDC vs. mDC + CN03). The variable assessed is the cell area depending on distance from the cell base after 60 minutes of incubation with theVLPs. Results correspond to one experiment in which we measured 7 control cells and 14 cells treated with CN03.  For the interaction between the column (treatment) and row (distance from cell base) we have: 6,52  % of total variation; p= 0,0706. For the column (treatment) factor we have: 2,327% of total variation; p = 0,0405. For the row factor (distance from cell base) we have: 20,36% of total variation; p <0,0001.  Multiple comparison’s Bonferroni test gave the following significant results:  Control mDC vs. mDC +CN03 (0-2um from cell base) Mean diff = -44,16; 95% CI = -86,48 to -1,833; p= 0,0355  **Supplementary Figure 1A**. Non parametric Mann Whitney test. We show the mean + SD of siglec-1 average cluster area per cell normalized to the values of in silico randomly distributed spots. We analyzed 8 iDC and 10 mDC from one representative experiment (out of 3 experiments from 3 different donors).  P value = 0.0266.  In this occasion we chose the non-parametric assumption due to the relative low number of cells analysed. Applying a parametric unpaired T-test the two-tailed significance would be higher P=0.019.  **Supplementary Figure 1A, 1B and 1C.** Non parametric Mann Whitney test. We show the mean + SD of siglec-1 average cluster area (A), NND between siglec-1 spots (B), and number of molecules per spot (C) per cell normalized to the corresponding values of in silico randomly distributed spots in mDC and iDC. We analyzed 6 cells per condition from one representative experiment (out of 3 experiments from 3 different donors).  Supplementary Figure 1A P value = 0.0266. 1B  In this occasion we chose the non-parametric assumption due to the relative low number of cells analysed. Applying a parametric unpaired T-test the two-tailed significance would be higher P=0.019.  **Supplementary Figure 2C:** Two way ANOVA matched values are spread in rows (number of molecules per spot), in the columns we have cell type (mDC vs. mDC+ CytoD). N=2 experiments. Experiment 1: 9 control cells vs 10 CytoD; Experiment 2: 10 control cells vs 10 CytoD. We calculated the frequency of molecules per spots (i.e 1, 2, 3, >4) pulling together all the clusters measured in all the cells analyzed (control vs. CK666) for each experiment.  We have a significant source of variation depending on the interaction between the row (number of molecules per spot) and the column (mDC vs. mDC +CytoD) factors: 12.54 % of total variation. P value = 0.0007  The significance presented between bars corresponds to a Bonferroni test in which we compare the mean value of number of molecules per spot frequency (i.e. 1, 2, 3 more than 4) in mDC vs. mDC + CytoD.  For 1 molecules per spot (mDC vs. mDC + CytoD): Mean diff = -0.2251; 95% CI = -0,3050 to -0,1451; p=0.0011  For 2 molecules per spot (iDC vs. mDC): Mean diff = 0.09905 1; 95% CI= 0,01912 to 0,1790; p=0.0236  **Supplementary Figure 2E:** Two way ANOVA matched values are spread in rows (control vs SMIFH2) in the columns we have average position of immobile trajectories vs. average position of mobile trajectories per cell. One experiment in which we analyzed 8 control and 10 SMIFH2 treated cells.  The significance presented between bars corresponds to a Bonferroni test in which we compare the average position of immobile trajectories vs. mobile trajectories in mDC vs. mDC + SMIFH2.  For mDC (compare average position of mobile trajectories vs. average position of immobile trajectories). Mean diff = 10,25; 95% CI = 0,9299 to 19,56; p=0.0303  For mDC + SMIFH2 (compare average position of mobile trajectories vs. average position of immobile trajectories). Mean diff = -2,512; CI = -10.85 to 5.822; p=0.9338  **Supplementary Figure 2I:** Two way ANOVA matched values are spread in rows (frequency of molecules per cluster) in the columns we have relative position in the cell (back vs. front). N=2. Experiment 1= 14 cells. Experiment 2 = 10 cells. The graphs show the mean + S.E.M of the average molecules per cluster frequency at the back and the front of all the cells analyzed.  The significance presented between bars corresponds to a Bonferroni test in which we compare the frequency of molecules per cluster at the back and the front of the cell  For 1 molecules per spot (back vs. front). Mean diff = -0,2478; 1; 95% CI = -0,4070 to -0,08863; P= 0,0043.  **Supplementary Figure 3A.** Non parametric Mann Whitney test. We show the mean intensity + SD of pERM in iDC and mDC. Results correspond to one representative experiment (N=2). Experiment 1 = 14 iDC and 9mDC. Experiment 2 = 10 iDC and 10mDC.  Experiment 1: P = 0.0086 two tailed. Results shown in the example figure  Experiment 2: P = 0.0147 two tailed.  **Supplementary Figure 3B**. Non parametric Mann Whitney test. We show the mean intensity + SD of pERM in control mDC and cells treated with CT04. Results correspond to one representative experiment (N=2). Experiment 1 = 10 mDC and 10mDC + CT04 (one outliner was removed; value > mean + 2 times SD). Experiment 2 = 10 mDC and 7 mDC + CT04.  Experiment 1: P = < 0.0001 two tailed. Results shown in the example figure  Experiment 2: P = 0.0007 two tailed.  **Supplementary Figure 3C.** Non parametric Mann Whitney test. We show the mean intensity + SD of pERM in control mDC and cells treated with Y27623. Results correspond to one representative experiment (N=2). Experiment 1 = 16 mDC and 22 mDC + Y27623. Experiment 2 = 10 mDC and 13 mDC + Y27623.  Experiment 1: P = 0.0016 two tailed. Results shown in paper  Experiment 2: P = 0.1151 two tailed.  **Supplementary Figure 3E.** Non parametric Mann Whitney test. We show the mean intensity + SD of pERM in control iDC and cells treated with CN03. Results correspond to one representative experiment (N=2). Experiment 1 = 10 iDC and 24 iDC + CN03. Experiment 1 = 10 iDC and 18 iDC + CN03.  Experiment 1: P = 0.0001 two tailed.  Experiment 2: P = 0.0072 two tailed.  **Supplementary Figure 3F:** Two way ANOVA matched values are spread in rows (distance from the cell base) in the columns we have relative value of siglec-1 intensity in iDC vs iDC + CN03. N=2. Experiment 1: 10 iDC and 17 iDC + CN03. Experiment 2 = 10 iDC and 24 iDC+ CN03. The graphs show the mean + S.E.M of siglec-1 relative siglec-1 intensity from the cell base.  We have a significant source of variation depending on the column factor (iDC vs. iDC + CN03): 39.61% of total variation; p= 0.0051. The stars in the graph refer to this parameter.  **Supplementary Figure 3G:** Two way ANOVA matched values are spread in rows (distance from the cell base) in the columns we have relative value of pERM intensity in iDC vs iDC + CN03. N=2. Experiment 1: 10 iDC and 19 iDC + CN03. Experiment 2 = 10 iDC and 24 iDC+ CN03. The graphs show the mean + S.E.M of siglec-1 relative siglec-1 intensity from the cell base.  We have a significant source of variation depending on the column factor (iDC vs. iDC + CN03): 71.52 % of total variation; p< 0.0001. The stars in the graph refer to this parameter.  The significance presented between bars corresponds to a Bonferroni test in which we compare the relative pERM intensity at diference distance from the cell base (i.e. 0 to 1.5µm, 1.5µm to 3µm) in iDC vs. iDC + CN03.  For 0 to 1.5µm (iDC vs. iDC + CN03): Mean diff = -0,5603; 95% CI = -0,7507 to -0,3699; p=0.0004  For 1.5µm to 3µm (iDC vs. iDC + CN03): Mean diff = -0,4643; 95% CI = -0,6547 to -0,2739; p=0.0009  For 3µm to 4.5µm (iDC vs. iDC + CN03): Mean diff = -0,3208; 95% CI -0,5112 to -0,1304; p=0.0053  For 4.5µm to 6µm (iDC vs. iDC + CN03): Mean diff = -0,3486; 95% CI -0,5390 to -0,1582; p=0.0036  For 6µm to 7.5µm (iDC vs. iDC + CN03): Mean diff = -0,3526; 95% -0,5430 to -0,1622; p= 0,0034  **Supplementary Figure 3H:** Two way ANOVA; matched values are spread in rows (number of molecules per spot), in the columns we have cell type (control iDC vs. iDC + CN03). N=2 experiments. In each individual experiment we calculated the average frequency of the number of molecules per spot. Experiment 1: 9 iDC and 13 iDC + CN03. Experiment 2: 10 iDC and 18 iDC + CN03.  We have a significant source of variation depending on the interaction between the row (number of molecules per spot) and the column (iDC vs. iDC + CN03) factors: 17.19 % of total variation. P value = 0.0001.  The significance presented between bars corresponds to a Bonferroni test in which we compare the mean value of number of molecules per spot frequency (i.e. 1, 2, 3 more than 4) in iDC vs. iDC + CN03.  For 1 molecules per spot (iDC vs. iDC + CN03): Mean diff = 0,2901 ; 95% CI = 0,2338 to 0,3463 ; p=0.0001.  For 2 molecules per spot (iDC vs. iDC + CN03): Mean diff = -0,2388; 95% CI = -0,2950 to -0,1825; p=0.0002.  **Supplementary Figure 3J.** Non parametric Mann Whitney test. We show the mean intensity + SD of pERM in control mDC and cells treated with NSC668394. Results correspond to one representative experiment (N=2). Experiment 1 = 15 mDC and 13 mDC + NSC668394. Experiment 2 = 10 mDC and 8 mDC + NSC668394.  Experiment 1: P = 0.0003 two tailed.  Experiment 2: P = 0.004 two tailed.  **Supplementary Figure 3K:** Two way ANOVA matched values are spread in rows (distance from the cell base) in the columns we have relative value ofm siglec-1 intensity in mDC vs mDC + NSC668394 . N=2. Experiment 1: 10 mDC and 9 mDC + NSC668394 . Experiment 2 = 15 mDC and 13 mDC+ NSC668394. The graphs show the mean + S.E.M of siglec-1 relative siglec-1 intensity from the cell base.  We have a significant source of variation depending on the column factor (mDC vs. mDC + NSC668394): 19.58 % of total variation; p = 0.0022. The stars in the graph refer to this parameter.  **Supplementary Figure 3L:** Two way ANOVA; matched values are spread in rows (number of molecules per spot), in the columns we have cell type (control mDC vs. mDC + NSC668394). N=2 experiments. In each individual experiment we calculated the average frequency of the number of molecules per spot. Experiment 1: 14 mDC and 10 mDC + NSC668394 . Experiment 2: 17 mDC and 14 mDC + NSC668394.  We have a significant source of variation depending on the interaction between the row (number of molecules per spot) and the column (iDC vs. iDC + CN03) factors: 17.19 % of total variation. P value = 0.0001.  The significance presented between bars corresponds to a Bonferroni test in which we compare the mean value of number of molecules per spot frequency (i.e. 1, 2, 3 more than 4) in mDC vs. mDC + NSC668394  For 1 molecules per spot (mDC vs. mDC + NSC668394): Mean diff = -0,2135; 95% CI = -0,3352 to -0,09176; p=0.0065.  **Supplementary Figure 3M.** Non parametric Mann Whitney test. We show the mean intensity + SD of pERM in control mDC and cells treated with SMIFH2. Results correspond to one representative experiment (N=2). Experiment 1 = 16 mDC and 16 mDC + SMIFH2. Experiment 1 = 10 mDC and 15 mDC + SMIFH2.  Experiment 1: P = 0.6887 two tailed (non significant). Representative experiment shown in the paper  Experiment 2: P = 0.0623 two tailed (non significant)  **Supplementary Figure 3N:** Two way ANOVA matched values are spread in rows (distance from the cell base) in the columns we have relative value of siglec-1 intensity in mDC vs mDC + SMIFH2. N=2. Experiment 1: 10 mDC and 12 mDC + SMIFH2. Experiment 2 = 15 mDC and 16 mDC+ SMIFH2. The graphs show the mean + S.E.M of siglec-1 relative siglec-1 intensity from the cell base.  We have a significant source of variation depending on the column factor (mDC vs. mDC + SMIFH2): 13.87 % of total variation; p = 0.0407. The stars in the graph refer to this parameter.  **Supplementary Figure 4B:** Non parametric Mann Whitney test. We show the mean intensity + SD of VLP in control mDC and cells treated with CT04. Results correspond to one representative experiment (N=2). Experiment 1 = 14 mDC and 15 mDC + CT04 (one outliner removed. Value > mean + 2 times SD). Experiment 2 = 14 mDC and 14 mDC + CT04.  Experiment 1: P = 0,0049 two tailed. Representative experiment shown in the paper.  Experiment 2: P = 0,0482 two tailed.  **Supplementary Figure 5A:** Two way ANOVA. In the rows we have time of incubation with VLPs (5 m minutes, 30 minutes and 60 minutes), in the columns we have cell type (iDC vs. mDC). The variable assessed is the Pearson colocalization coefficient of siglec-1 with VLPs. Results correspond to one experiment in which we measured 12 iDC and 9 mDC at 5 minutes of incubation with VLPs, 10 iDC and 10 mDC at 30 minutes of incubation with VLPs and 12 iDC and 12 mDC at 60 minutes of incubation with VLPs  We have a significant source of variation depending on the column factor (iDC vs.mDC): 13,4 % of total variation; p=0,0003  We have a significant source of variation depending on the row factor (5 minutes vs. 30 minutes vs. 60 minutes): 34,26 % of total variation; p< 0.0001.  The significance presented between bars corresponds to a Bonferroni test in which we compare siglec-1 colocalization with VLPs at different times of incubation in iDC and mDC:  For iDC vs mDC (60 minutes) : Mean diff = -0,2327; 95% CI = -0,4148 to -0,05049; p=0,0078  **Supplementary Figure 5B:** Two way ANOVA. In the rows we have time of incubation with VLPs (30 minutes and 60 minutes), in the columns we have cell type (iDC vs. mDC). The variable assessed is the mean area of siglec-1 colocalizing spots per cell (normalized to the average value obtained after 5 minutes of incubation with VLPs). Results correspond to one experiment in which we measured 10 iDC and 10 mDC at 30 minutes of incubation with VLPs and 10 iDC and 9 mDC at 60 minutes of incubation with VLPs  We have a significant source of variation depending on the column factor (iDC vs.mDC): 56,17 % of total variation; p <0,0001    We have a significant source of variation depending on the row factor (30 minutes vs. 60 minutes): 12,53 % of total variation; p= 0,0008.  The significance presented between bars corresponds to a Bonferroni test in which we compare the area of siglec-1 spots colocalizing with VLPs at different times of incubation (30 minutes and 60 minutes)  For 30 minutes vs. 60 minutes (iDC) : Mean diff = -0,1819; 95% CI = -0,3066 to -0,05715; p= 0,0033  For iDC vs. mDC (30 minutes) : Mean diff = -0,3408; 95% CI = -0,4655 to -0,2161; p <0,0001  For iDC vs. mDC (60 minutes) : Mean diff = -0,2617; 95% CI = -0,3940 to -0,1294; p= 0,0001  **Supplementary Figure 5C:** Two way ANOVA. In the rows we have time of incubation with LUVs or VLPs (30 minutes and 60 minutes), in the columns we have the type of siglec-1 binding particles assessed (VLPs, LUV with 4% GM-1 and LUV with 0.5% GM1). The variable assessed is the average number of siglec-1 molecules per spot not colocalizing with either VLPs or LUVs per cell. Results correspond to one experiment in which we measured 10 cells pulsed for 30 minutes with VLPs, 10 cells pulsed for 60 minutes with VLPs, 14 cells pulsed for 30 minutes with LUVs 4% GM-1, 15 cells pulsed for 60 minutes with LUVs 4% GM-1, 16 cells pulsed for 30 minutes with LUVs 0.5 % GM-1, 14 cells pulsed for 60 minutes with LUVs 0.5 % GM-1.  We have a significant source of variation depending on the interaction between the column factor (type of particle) and the row factor (time of incubation): 16,28% of total variation; p = 0,0002  We have a significant source of variation depending on the row factor (time of incubation): 13,70% of total variation;p = 0,0001  We have a significant source of variation depending on the column factor (type of particle): 7,140 % of total variation; p = 0,0164.  The significance presented between bars corresponds to a Bonferroni test in which we compare the average number of molecules per spot per cell colocalizing with either VLPs or LUVs (0.5% and 4% GM-1) at different times of incubation (30 minutes vs. 60 minutes).  For VLP vs 4% GM-1 LUV (30 minutes) : Mean diff = -0,07534 ; 95% CI = -0,2962 to 0,1455; p >0,9999  For VLP vs 0.5% GM-1 LUV (30 minutes) : Mean diff = 0,2983 ; 95% CI = 0,08050 to 0,5161; p =0,0038  For 4% vs 0.5% GM-1 LUV (30 minutes) : Mean diff = 0,3736 ; 95% CI = 0,1754 to 0,5719; p <0,0001  For VLP vs 4% GM-1 LUV (60 minutes) : Mean diff = -0,1673; 95% CI = -0,3851 to 0,05046; p = 0,1910  For VLP vs 0.5% GM-1 LUV (60 minutes) : Mean diff = -0,2102 ; 95% CI = -0,4346 to 0,01421; p = 0,0738  For 4% vs 0.5% GM-1 LUV (60 minutes) : Mean diff = -0,04285 ; 95% CI = -0,2450 to 0,1593; p = >0,9999  **Supplementary Figure 6A:** Two way ANOVA. In the columns we have time of incubation with VLPs (5 minutes, 30 minutes and 60 minutes), in the rows we have distance from the cell base. The variable assessed is the colocalization between siglec-1 and VLP (Manders coefficient). For the times 5 minutes and 30 minutes we performed 3 independent experiments, for the 60 minutes point we performed 2 independent experiments. Experiment 1, 2, 3 and 4 (5 minutes): average of 9, 13 and 15 cells. Experiment 1, 2, 3 and 4 (30 minutes): average of 15, 13 and 13 cells. Experiment 1, 2 (60 minutes): average of 11 and 11 cells.  We have a significant source of variation depending on the row factor (distance from the cell base): 50,93 % of total variation; p <0,0001  Multiple comparison Bonferroni test in which we compared the colocalization between siglec-1 and VLP depending on time of incubation and distance from the cell base.  For 30 minutes vs 5 minutes (0-2um from the cell base) : Mean diff = 0,1117; 95% CI = 0,002780 to 0,2206; p = 0,0429  **Supplementary Figure 6B:** Two way ANOVA. In the columns we have time of incubation with 4% GM-1 LUVs (5 minutes, 30 minutes and 60 minutes), in the rows we have distance from the cell base. The variable assessed is the colocalization between siglec-1 and LUV (Manders coefficient). Results correspond to one experiment in which we measured 15 cells for the 5 minutes time, 7 cells for the 30 minutes time and 13 cells for the 60 minutes time.  We have a significant source of variation depending on the interaction between the row factor (distance from the cell base) and the column factor (time of incubation) : 7,430 % of total variation; p = 0,0058  We have a significant source of variation depending on the row factor (distance from the cell base): 36,65% of total variation; p <0,0001  Multiple comparison Bonferroni test in which we compared the colocalization between siglec-1 and LUV depending on time of incubation and distance from the cell base.  For 5 minutes vs 60 minutes (0-2um from the cell base) : Mean diff = -0,1663; 95% CI = -0,2610 to -0,07163; p = 0,0001  **Supplementary Figure 6D** . Non parametric Mann Whitney test. We show the mean intensity of pMLC in mDC at steady-state and after 60 minutes of incubation with VLPs. Results from one experiment in which we analyzed 7 control cells in the absence of VLP and 8 cells after 60 minutes incubation with VLPs (one outliner removed. Value > mean + 2 times SD).  Two-taile p-value= 0,0041  **Supplementary Figure 6E** .Ratio paired t-test. We show the ratio of p-cofilin/cofilin measured by WB in mDC incubated for different times with VLPs. state and after 60 minutes of incubation with VLPs. N=4.  No VLPs vs 5 minutes VLP: two-tailed p-value = 0,1089  No VLPs vs 30 minutes VLP: two-tailed p-value = 0,0310  No VLPs vs 60 minutes VLP: two-tailed p-value = 0,0754  5 minutes VLPs vs 30 minutes VLP: two-tailed p-value = 0,0064  5 minutes VLPs vs 60 minutes VLP: two-tailed p-value = 0,4740  30 minutes VLPs vs 60 minutes VLP: two-tailed p-value = 0,9292 |  |
|  |  |  |
| **Data availability** | **Indicate where provided: section/submission form** | **N/A** |
| For newly created and reused datasets, the manuscript includes a data availability statement that provides details for access (or notes restrictions on access). |  | x |
| When newly created datasets are publicly available, provide accession number in repository OR DOI and licensing details where available. |  | x |
| If reused data is publicly available provide accession number in repository OR DOI, OR URL, OR citation. |  | x |
|  |  |  |
| **Code availability** | **Indicate where provided: section/figure legend** | **N/A** |
| For any computer code/software/mathematical algorithms essential for replicating the main findings of the study, whether newly generated or re-used, the manuscript includes a data availability statement that provides details for access or notes restrictions. |  | x |
| Where newly generated code is publicly available, provide accession number in repository, OR DOI OR URL and licensing details where available. State any restrictions on code availability or accessibility. |  | x |
| If reused code is publicly available provide accession number in repository OR DOI OR URL, OR citation. |  | x |

**Reporting:**

The MDAR framework recommends adoption of discipline-specific guidelines, established and endorsed through community initiatives.

| **Adherence to community standards** | **Indicate where provided: section/figure legend** | **N/A** |
| --- | --- | --- |
| State if relevant guidelines (e.g., ICMJE, MIBBI, ARRIVE, STRANGE) have been followed, and whether a checklist (e.g., CONSORT, PRISMA, ARRIVE) is provided with the manuscript. |  | x |

* We provide the following guidance regarding transparent reporting and statistics; we also refer authors to [Ten common statistical mistakes to watch out for when writing or reviewing a manuscript](https://doi.org/10.7554/eLife.48175).

**Sample-size estimation**

- You should state whether an appropriate sample size was computed when the study was being designed
- You should state the statistical method of sample size computation and any required assumptions
- If no explicit power analysis was used, you should describe how you decided what sample (replicate) size (number) to use

**Replicates**

- You should report how often each experiment was performed
- You should include a definition of biological versus technical replication
- The data obtained should be provided and sufficient information should be provided to indicate the number of independent biological and/or technical replicates
- If you encountered any outliers, you should describe how these were handled
- Criteria for exclusion/inclusion of data should be clearly stated
- High-throughput sequence data should be uploaded before submission, with a private link for reviewers provided (these are available from both GEO and ArrayExpress)

**Statistical reporting**

- Statistical analysis methods should be described and justified
- Raw data should be presented in figures whenever informative to do so (typically when N per group is less than 10)
- For each experiment, you should identify the statistical tests used, exact values of N, definitions of center, methods of multiple test correction, and dispersion and precision measures (e.g., mean, median, SD, SEM, confidence intervals; and, for the major substantive results, a measure of effect size (e.g., Pearson's r, Cohen's d)
- Report exact p-values wherever possible alongside the summary statistics and 95% confidence intervals. These should be reported for all key questions and not only when the p-value is less than 0.05.

**Group allocation**

- Indicate how samples were allocated into experimental groups (in the case of clinical studies, please specify allocation to treatment method); if randomization was used, please also state if restricted randomization was applied
- Indicate if masking was used during group allocation, data collection and/or data analysis
